# A genome‐wide association study of the frailty index highlights brain pathways in ageing

**DOI:** 10.1111/acel.13459

**Published:** 2021-08-25

**Authors:** Janice L. Atkins, Juulia Jylhävä, Nancy L. Pedersen, Patrik K. Magnusson, Yi Lu, Yunzhang Wang, Sara Hägg, David Melzer, Dylan M. Williams, Luke C. Pilling

**Affiliations:** ^1^ Epidemiology and Public Health Group University of Exeter Medical School Exeter UK; ^2^ Department of Medical Epidemiology and Biostatistics Karolinska Institutet Stockholm Sweden; ^3^ Department of Psychology University of Southern California Los Angeles CA USA; ^4^ Center on Aging University of Connecticut Farmington CT USA; ^5^ MRC Unit for Lifelong Health and Ageing at UCL University College London London UK

**Keywords:** ageing, frailty, frailty index, genetics, UK Biobank

## Abstract

Frailty is a common geriatric syndrome and strongly associated with disability, mortality and hospitalization. Frailty is commonly measured using the frailty index (FI), based on the accumulation of a number of health deficits during the life course. The mechanisms underlying FI are multifactorial and not well understood, but a genetic basis has been suggested with heritability estimates between 30 and 45%. Understanding the genetic determinants and biological mechanisms underpinning FI may help to delay or even prevent frailty. We performed a genome‐wide association study (GWAS) meta‐analysis of a frailty index in European descent UK Biobank participants (*n *= 164,610, 60–70 years) and Swedish TwinGene participants (*n *= 10,616, 41–87 years). FI calculation was based on 49 or 44 self‐reported items on symptoms, disabilities and diagnosed diseases for UK Biobank and TwinGene, respectively. 14 loci were associated with the FI (*p *< 5*10^−8^). Many FI‐associated loci have established associations with traits such as body mass index, cardiovascular disease, smoking, HLA proteins, depression and neuroticism; however, one appears to be novel. The estimated single nucleotide polymorphism (SNP) heritability of the FI was 11% (0.11, SE 0.005). In enrichment analysis, genes expressed in the frontal cortex and hippocampus were significantly downregulated (adjusted *p *< 0.05). We also used Mendelian randomization to identify modifiable traits and exposures that may affect frailty risk, with a higher educational attainment genetic risk score being associated with a lower degree of frailty. Risk of frailty is influenced by many genetic factors, including well‐known disease risk factors and mental health, with particular emphasis on pathways in the brain.

## Graphical abstract

This genome‐wide association study meta‐analysis of the frailty index (FI) in UK Biobank and TwinGene, identified 14 loci associated with the FI. Many FI‐associated loci have established associations with well‐known disease risk factors such as BMI, cardiovascular disease, smoking, HLA proteins, depression and neuroticism. However 1 was novel. Risk of frailty is influenced by many genetic factors, including well‐known disease risk factors and mental health, with particular emphasis on pathways in the brain.

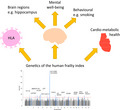

## 1 INTRODUCTION

Frailty is a common geriatric syndrome involving multi‐system impairment, which is associated with increased vulnerability to stressors (Dodds & Sayer, 2016). Frailty is a major public health issue in geriatric populations and is becoming increasingly common with ageing demography (Morley, 2016). Many frailty definitions exist but two of the most commonly used are the frailty phenotype (FP) (Fried et al., 2001) and the frailty index (FI) (Mitnitski et al., 2001). The FP is a clinical syndrome based on the presence of three of five physical components (exhaustion, weakness, slow walking speed, unintentional weight loss and low physical activity), whereas the FI is based on the accumulation of a number of health deficits during the life course. While both measures are related to adverse ageing outcomes, the two instruments are differently orientated and the underlying physiological and biological differences between the FP and FI are important (Cesari et al., 2014) (Cesari et al., 2017). The FI may be better at discriminating at the lower to middle end of the frailty continuum, making it better suited for measuring frailty at younger age (Blodgett et al., 2015).

Higher FI values are associated with many negative health outcomes including disability, mobility limitations, a variety of chronic diseases and hospitalization, and mortality (Williams et al., 2019) (Kojima et al., 2018) (Vermeiren et al., 2016). The prevalence of the FI is estimated as 18% worldwide in community‐dwelling older adults aged 60 and above (Siriwardhana et al., 2018). The mechanisms underlying the FI are likely multifactorial but not well understood. Studies have suggested a genetic basis, with heritability estimates between 30 and 45% (Livshits et al., 2018) (Young et al., 2016) (Kim et al., 2013). Candidate gene association studies for the FI have suggested the involvement of genes in inflammatory pathways, including interleukin‐18 (Mekli et al., 2015). A first GWAS of the FI included two representative samples from the United States and U.K. (the discovery sample: the Health and Retirement Study, *n* = 8532; and the replication sample: the English Longitudinal Study of Ageing, *n* = 5248), and identified two SNPs associated with FI variation (Mekli et al., 2018). rs6765037 (located near the gene *KBTBD12*) was associated in the discovery sample only, and rs7134291 (located in *GRIN2B*)—which plays a role in brain development, synaptic plasticity and cognition—showed a nominal replication. Further large‐scale GWAS are needed to corroborate these findings and to more thoroughly understand the genetic determinants of frailty and associated biological pathways. These might provide insights for interventions to prevent and delay frailty and hence promote healthier and more independent ageing.

We undertook a GWAS meta‐analysis of frailty, measured using the FI, in 60‐ to 70‐year‐old participants of European descent from the UK Biobank cohort and Swedish TwinGene participants aged 41 to 87 years. To gain insights into the functional relevance of emergent genetic associations, we examined the relationship of the frailty risk loci with circulating proteins and tissue‐specific gene expression and epigenetic profiles. We also leveraged the GWAS findings in Mendelian randomization analyses to identify modifiable physiological, lifestyle or environmental traits that could be targeted by clinical and/or public health interventions to mitigate frailty risk.

## 2 RESULTS

### 2.1 Study characteristics

In this GWAS meta‐analysis of UK Biobank and TwinGene, we included 164,610 UK Biobank participants of European descent aged 60 to 70 years at baseline (mean 64.1, SD 2.8), which included 84,819 females (51.3%). The FI score ranged from zero to 27, from 49 deficits in total, and the mean proportion of deficits was 0.129 (SD 0.075), with a slightly higher mean score in females (0.132, SD 0.076) compared to males (0.125, SD 0.074) (Table 1). The TwinGene participants (*n* = 10,616) were Swedish nationals aged 41 to 87 years (mean 58.3, SD 7.9) with 5,577 females (52.5%), and all of European descent. The TwinGene FI score ranged from zero to 26.25 deficits (44 considered in total), and the mean proportion of deficits was 0.121 (SD 0.080).

**TABLE 1:** Baseline characteristics of study populations

	UK Biobank	TwinGene	SATSA
N	164,610	10,616	368
Females, n (%)	84,819 (51.3)	5,577 (52.5)	223 (60.6)
Age, mean (sd)	64.1 (2.8)	58.3 (7.9)	68.6 (9.6)
Frailty Index, mean (sd)*	0.129 (0.075)	0.121 (0.080)	0.1 (0.087)
Frailty Index, range	0–27	0–26.25	0–19
Number of items used to compose FI	49	44	42

SATSA = Swedish Adoption/Twin Study of Aging.

* Proportion of deficits.

The Swedish Adoption/Twin Study of Aging (SATSA) participants (*n*=368)—whose data were used in DNA methylation‐related follow‐up analyses—were all of European descent, aged 48 to 93 years (mean 68.6, SD 9.6), with 223 (60.6%) females. The SATSA FI score ranged from zero to 19 deficits (42 considered in total), and the mean proportion of deficits was 0.100 (SD 0.087).

### 2.2 GWAS meta‐analysis of the FI

**FIGURE 1:**
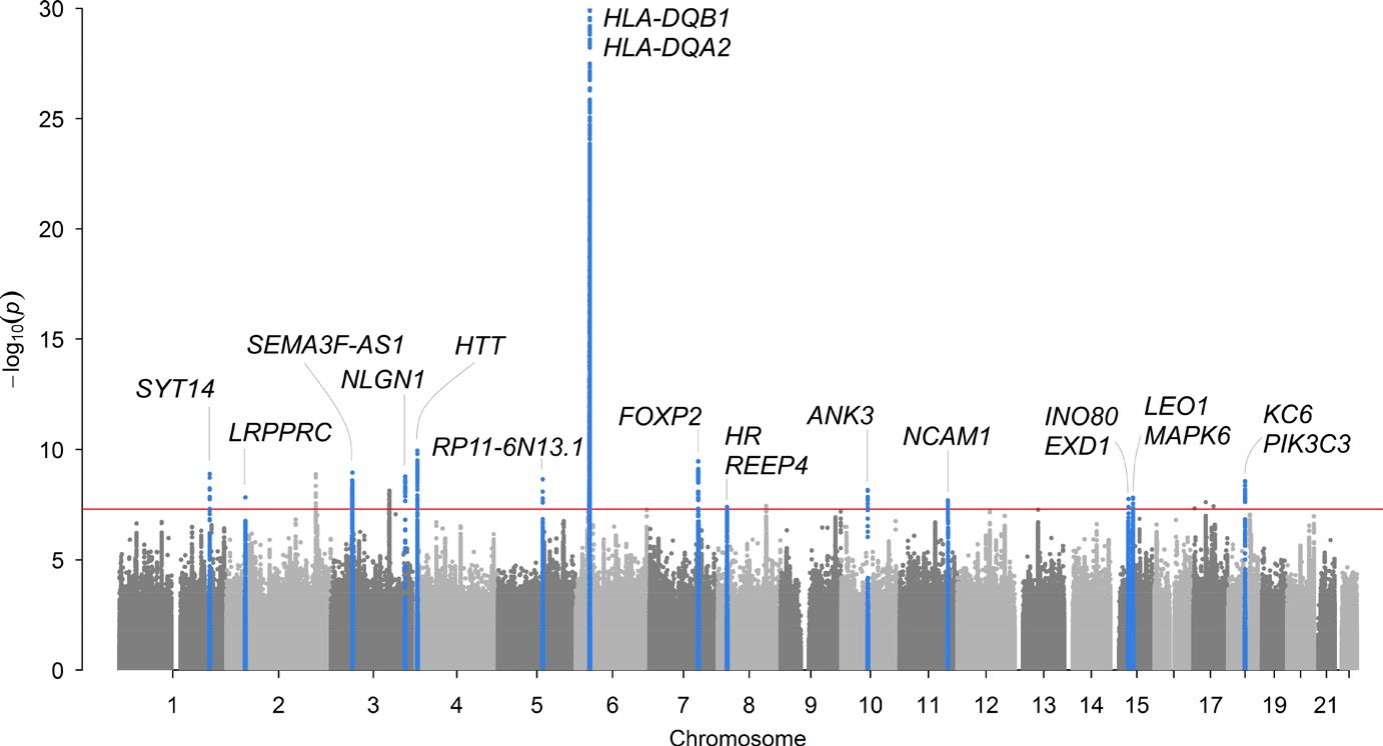
Manhattan plot for genome‐wide association study of Frailty Index. Meta‐analysis GWAS of Frailty Index (normalized) in 164,610 UK Biobank participants aged 60–70 of European descent and 10,616 TwinGene participants aged 41–87 years. Primary analysis included 7,666,890 autosomal variants with minor allele frequency (MAF) >0.1%. Hardy–Weinberg p‐value >1x10^−9^ and imputation quality >0.3 in both cohorts. Linear mixed‐effects regression models (BOLT‐LMM software (Loh et al., 2015), which accounts for relatedness and population structure), were adjusted for age, sex, assessment centre (22 categories) and genotyping array (2 categories: Axiom or BiLEVE). There are 14 loci associated with p<5*10^−8^ (red line) in the meta‐analysis, highlighted in blue. In secondary analysis of 8,828,853 variants only available in UK Biobank, 6 additional loci were associated p<5*10^−8^ (plotted but not highlighted). Genes are those nearest to the lead variants. See Table 2 for primary meta‐analysis results. See Tables S1 and S2 for full details

We identified 14 loci associated (*p* < 5*10^−8^) with the FI (*n *= 2,007associated variants in total) in the meta‐analysis of UK Biobank participants and TwinGene (Figure 1, Table 2 and Table S1 for further details). All lead variants were imputed with high accuracy (≥90%), and there was no evidence for deviation from Hardy–Weinberg equilibrium (*p* > 0.01). The Lambda GC (genomic control) value was high (1.32; see Figure S1 for QQ plot); however, the intercept from Linkage Disequilibrium Score Regression (LDSR) analysis was 1.02 (SE 0.009), indicating that the inflation in test statistics is mainly due to polygenicity (many variants with small effects on frailty), and the bias potentially due to population stratification is minimal (Bulik‐Sullivan et al., 2015). The single nucleotide polymorphism (SNP)‐based heritability (*h^2^_g_*) of the FI was estimated to be 0.11 (SE 0.005), that is 11%, by LDSR. Full GWAS summary statistics are available to download from the GWAS catalog (https://www.ebi.ac.uk/gwas/downloads/summary‐statistics; study accession GCST90020053) or directly from (https://doi.org/10.6084/m9.figshare.9204998). LocusZoom plots for each of the 14 loci are shown in Figures S2A‐N.

**TABLE 2:** GWAS meta‐analysis associations with Frailty Index in UK Biobank and TwinGene

Gene	RSID	CHR:BP	A1:A2	A1F	BETA	SE	*p*	D	HetP	BETA2	SE2	Known signal from GWAS catalog
‐ Meta‐analysis												
*SYT14*	rs12739243	1:210302043	T:C	0.78	0.024	0.004	1.3E−09	++	0.17	0.091	0.015	Smoking initiation
*LRPPRC*	rs4952693	2:44151808	T:C	0.37	−0.019	0.0034	1.5E−08	‐‐	0.43	−0.066	0.013	~Hand grip strength
lncRNA (*SEMA3F*‐*AS1*)	rs2071207	3:50159844	T:C	0.52	0.019	0.0033	1.5E−08	++	0.47	0.069	0.012	Intelligence, HDL, BMI, Inflammatory bowel disease…
*NLGN1*	rs583514	3:173114167	T:C	0.49	−0.020	0.0033	1.7E−09	‐+	0.04	−0.074	0.012	BMI, longevity, smoking initiation, WBC
*HTT*	rs82334	4:3225371	A:C	0.68	0.022	0.0035	3.1E−10	++	0.18	0.086	0.013	Waist‐hip ratio, WBC, ~eGFR, ~LDL, ~longevity…
x..[lncRNA]	rs1363103	5:103917837	T:C	0.62	0.019	0.0034	2.2E−08	+‐	0.08	0.078	0.013	Subjective wellbeing, ~BMI, ~depression, ~grip strength…
*[HLA‐DQB1]..x..[HLA‐DQA2]*	rs9275160	6:32652620	A:G	0.34	0.038	0.0035	7.2E−28	++	0.03	0.152	0.013	~Asthma, ~Rheumatoid arthritis, ~Urate levels…
*FOXP2*	rs2396766	7:114318071	A:G	0.47	0.020	0.0033	1.2E−09	+‐	0.07	0.078	0.012	~BMI, ~Household income, ~Smoking initiation…
*[HR].x..[REEP4]*	rs56299474	8:21992804	A:C	0.17	0.024	0.0044	3.9E−08	++	0.41	0.087	0.016	*
*ANK3*	rs4146140	10:61885362	T:C	0.38	−0.020	0.0034	6.8E−09	‐‐	0.67	−0.067	0.013	~BMI
*NCAM1*	rs10891490	11:112885527	T:C	0.41	0.019	0.0034	2.0E−08	++	0.37	0.066	0.013	Depressive symptoms, Neuroticism, Smoking initiation…
*[INO80]..x. [EXD1]*	rs3959554	15:41443924	A:G	0.58	−0.019	0.0034	1.7E−08	‐‐	0.36	−0.070	0.013	Age at menopause, Height, ~Asthma, ~eGFR, ~SBP…
*[LEO1]x*..*[MAPK6]*	rs17612102	15:52264094	T:C	0.41	−0.019	0.0034	2.8E−08	‐‐	0.67	−0.072	0.013	~BMI, ~Reticulocyte volume
*[KC6]*…*x*…*[PIK3C3]*	rs8089807	18:39322639	T:C	0.19	−0.025	0.0043	6.5E−09	‐‐	0.26	−0.099	0.016	Neuroticism, Education, ~Smoking initiation
‐ UK Biobank only												
*[KLF7]x..[MYOSLID‐AS1]*	rs111432129	2:208040003	A:G	0.70	−0.022	0.0037	1.3E−09			−0.084	0.014	Insomnia, ~Brain region volumes, ~Self‐rated health
*STAG1*	rs758591652	3:136138073	TTTTC:T	0.24	0.023	0.004	7.3E−09			−0.086	0.015	Height, Intelligence, Intraocular pressure, Neuroticism…
*CSMD3*	rs796921150	8:113025459	G:GA	0.50	0.019	0.0034	3.5E−08			0.063	0.013	*
*PAFAH1B1*	rs7219015	17:2555592	T:C	0.22	0.023	0.0041	4.6E−08			0.084	0.015	~Education, ~Neuroticism, ~Subjective wellbeing
*CRLF3*	rs77338984	17:29142814	A:G	0.10	−0.032	0.0058	2.4E−08			−0.120	0.021	BMI, Haemoglobin concentration
*[ZNF652]..x.[PHB]*	rs10625032	17:47472852	T:TTTC	0.75	0.022	0.004	3.7E−08			0.082	0.015	~Asthma

*Abbreviations*: Gene = nearest gene to variant x (intronic or exonic if just gene name), RSID = variant identifier, CHR:BP = Genomic position (b37), A1:A2 = effect allele: other allele, A1F = A1 allele frequency, BETA = A1 effect on FI (quantile normalized), SE = standard error, P = p‐value, D = direction of effect in UK Biobank and TwinGene, HetP = heterogeneity p‐value from meta‐analysis, BETA2 = A1 effect on FI (total points, untransformed) in UK Biobank only, SE2 = SE for BETA2 (UK Biobank only). See Table S1 for further details.

*Known signal from GWAS catalog* (downloaded 12 April 2021): either the lead SNP or a variant in high LD (R2>0.8) is associated with the trait. ~ indicates a variant in moderate LD (R2>0.4 and <0.8) is associated with the indicated trait. *Does not appear in the GWAS catalog. See Table S2 for full mapping to GWAS catalog (with complete references).

Of the 14 signals identified above the threshold for genome‐wide significance, two were significant below the more robust *p*‐value threshold of 1*10^−9^: rs9275160 (nearest genes *HLA*‐*DQB1* and *HLA*‐*DQA2*) and rs82334 (nearest gene *HTT)*. Two of the 14 loci (rs583514 in *NLGN1* and rs2396766 in *FOXP2*) had significant heterogeneity between the studies (effect allele associated with FI in opposite directions), so should be interpreted with caution (Table 2).

Many of the loci (13 of the 14) have previously been associated with traits and diseases in the GWAS Catalog (Buniello et al., 2019) (as of 12 April 2021) such as body mass index (BMI), cardiovascular disease, smoking initiation, HLA proteins, depression and neuroticism (Table 2, Table S2). For some, it is the lead SNP itself identified in another GWAS, whereas for others it is a variant in partial LD that has appeared previously (e.g. rs4952693 in gene *LRPPRC* is associated with FI in our analysis, with a correlated variant rs10186876 *R* ^2^ = 0.61 known to be associated with handgrip strength (Willems et al., 2017); see Table 2 and Table S2 for details). One of the associations appears to be novel: rs56299474 (*p* = 4*10^−8^) is located between genes *HR* and *REEP4* and has not previously appeared in the GWAS catalog. However, it is a single associated variant with no other variants at the locus showing appreciable evidence of association, so further evidence is required for a robust conclusion.

In separate analysis of variants available only in UK Biobank (not imputed in TwinGene), we identified six further loci that require additional validation (Table 2). Five of the six have previously appeared in the GWAS catalog, with rs796921150 in gene *CSMD3* appearing in GWAS for the first time.

### 2.3 Candidate gene association analysis

Based on prior evidence we also specifically looked up variants for which we hypothesized an association with the FI a priori. First, the previous GWAS of FI (Mekli et al., 2018) identified 2 variants; however, neither were associated with FI: rs6765037 (*p *= 0.410), although rs7134291 was nominally significant (*p *= 0.018). We also investigated 22 variants associated with parents’ survival in three previous GWAS (Pilling et al., 2016) (Wright et al., 2019) (RHJ Timmers et al., 2019: of note, rs429358 (*APOE* e4)—related to both cardiovascular and Alzheimer's diseases—was not associated with the FI (*p* = 0.29) and the 9p21.3 variant rs2891168 in the lncRNA ANRIL—also known as *CDKN2B*‐*AS1*—showed evidence of association, although did not reach genome‐wide significance (*p* = 2*10^−4^). rs61348208 is associated with parents’ lifespan (RHJ Timmers et al., 2019) and is close to significance in this analysis (*RBM6* locus, *p* = 3*10^−7^) though still not reaching the genome‐wide cut‐off (5*10^−8^). See Table S2 for full results list.

### 2.4 Tissue enrichment analysis implicates brain pathways

FUMA (Functional mapping and annotation of genetic associations (Watanabe et al., 2017)) analysis of differentially expressed genes using tissue‐specific gene expression data (GTEx v8) identified four tissues with significant (multiple testing adjusted *p *< 0.05) downregulation of genes associated with FI: Brain (Frontal Cortex BA9), Brain (Cerebellar Hemisphere), Brain (Spinal cord cervical c‐1) and Brain (Hippocampus) (See Table S3). With gene‐set enrichment analysis using MAGMA software (in FUMA), we found nine pathways significantly enriched in the GWAS results for FI after adjustment for multiple testing (Bonferroni), including: MHC Class II complex; Translocation of Zap70 to immunological synapse, and asthma (*p *< 3*10^−6^; see Table S4 for details). In stratified linkage disequilibrium score regression (LDSR) analysis (Finucane et al., 2018), which identifies whether GWAS statistics are associated with specific gene‐tissue pairings in expression or chromatin modification, we found enrichment for the genetic determinants of frailty in several brain regions, including the hippocampus and frontal cortex (see Tables S5 and S6 for details).

### 2.5 GWAS hits as molecular quantitative trait loci (QTLs)

We examined whether there are established associations of the top 14 SNPs with concentrations of circulating proteins, metabolites, gene expression and/or epigenetic markers, as reported by previous QTL GWAS with genome‐wide significance (*p* < 5*10–8). Overall, 13 of the SNPs were associated with at least one molecular marker (Table S7), whereas no evidence for associations was identified for rs8089807. Three SNPs are established protein QTLs, with one variant (rs2071207) showing associations with concentrations of five distinct proteins. Nine of the SNPs were found to be expression QTLs, associated with mRNA varying from one to 47 genes, in up to 48 tissue types. The most widespread associations with expression were present for rs2071207, which is located in the sequence for the long non‐coding RNA *SEMA3F*‐*AS1* (adjacent to gene *RMB6*) on chromosome 3. In epigenetic marker data, 13 SNPs were identified as methylation QTLs in one or more blood cell types, with CpG associations per SNP ranging from one to 98. Additionally, five of these SNPs are associated with two types of histone modification (H3K27ac and H3K4me1) in blood cells, including an influence of rs2071207 on both. Variant rs2071207 was the only SNP with evidence of being a splicing QTL, with effects on *RMB6* transcript length. No associations were identified for any SNP with metabolites.

### 2.6 Methylation—FI associations

To identify whether risk variants could be related to frailty via DNA methylation differences, we assessed whether the FI‐associated genetic variants harboured methylation quantitative trait loci (mQTL) and whether methylation levels in such loci demonstrated further associations with the FI in SATSA. Of the total 2,007 variants associated with the FI, 1,573 were cis‐methylation quantitative trait loci (cis‐mQTL) identified in SATSA. These 1,573 variants had 12,081 demonstrable associations with CpG sites in total; single variants were commonly associated with multiple CpG sites, and there were 103 unique CpGs among all associations. Associated variants for the 103 CpGs are shown in Table S8. Methylation level in one (cg20614157) of the 103 CpG sites was significantly associated with the SATSA FI after Bonferroni correction (see Table 3; full results for all 103 sites shown in Table S9), and 6 CpG sites were associated with a nominal *p* < 0.05.

**TABLE 3:** Association between methylation levels in mQTL‐associated CpGs and Frailty Index in SATSA

CpG site	Estimate	SE	*p*	*p* ^Bonferroni^	CHR	BP	UCSC Ref Gene	Associated variant
cg20614157	9.04E−04	0.0003	<0.001	0.037	6	31980845	*TNXB,TNXA,STK19*	rs17421133, rs2077116, rs17207951, rs2857009, rs2071295, rs17201588, rs6902493, rs41268896
cg19376858	8.38E−04	0.0003	0.008	0.084	6	31980856	*TNXB,TNXA,STK19*	rs6449, rs3020644, rs9267806, rs4713506, rs6941112, rs4713505, rs9267803, rs6463, rs9267802, rs4151657, rs8111, rs17421133, rs2280774, rs6415128, rs2894250, rs2228628, rs2857009, rs2077116, rs6902493, rs17201588
cg15321244	−2.23E−02	0.0088	0.011	1	6	32729643	*HLA‐DQB2*	rs9275987, rs2261566
cg23928032	−9.75E−03	0.0038	0.011	1	6	31964391	*C4B.C4A*	rs9267806, rs4713506, rs4713505, rs9267803, rs6463, rs9267802, rs8111, rs17421133, rs2228628, rs2857009, rs2077116, rs6902493, rs17201588, rs17207951, rs2239689, rs28361052, rs2071295, rs17421624, rs41268896, rs2071293
cg01937212	−1.43E−02	0.0062	0.020	1	6	32295097	*C6orf10*	rs9268301, rs9368714, rs2076540, rs2746115, rs2143466, rs761187, rs9268129, rs471081, rs6929776, rs9348880, rs2022533, rs6457544, rs6457543, rs565571, rs9366793, rs9394087, rs9268141, rs546857, rs477005, rs557539
								
cg11391305	−1.43E−02	0.007	0.042	1	6	32731438	*HLA‐DQB2*	rs9275987

Abbreviations: BP = Genomic position (b37);CHR = chromosome; mQTL = methylation quantitative trait loci; SATSA = Swedish Adoption/Twin Study of Aging; SE = standard error.

* If >20 associated variants, the first 20 with lowest p‐values are included (see Table S9 for full results).

### 2.7 Mendelian Randomization (MR)

**FIGURE 2:**
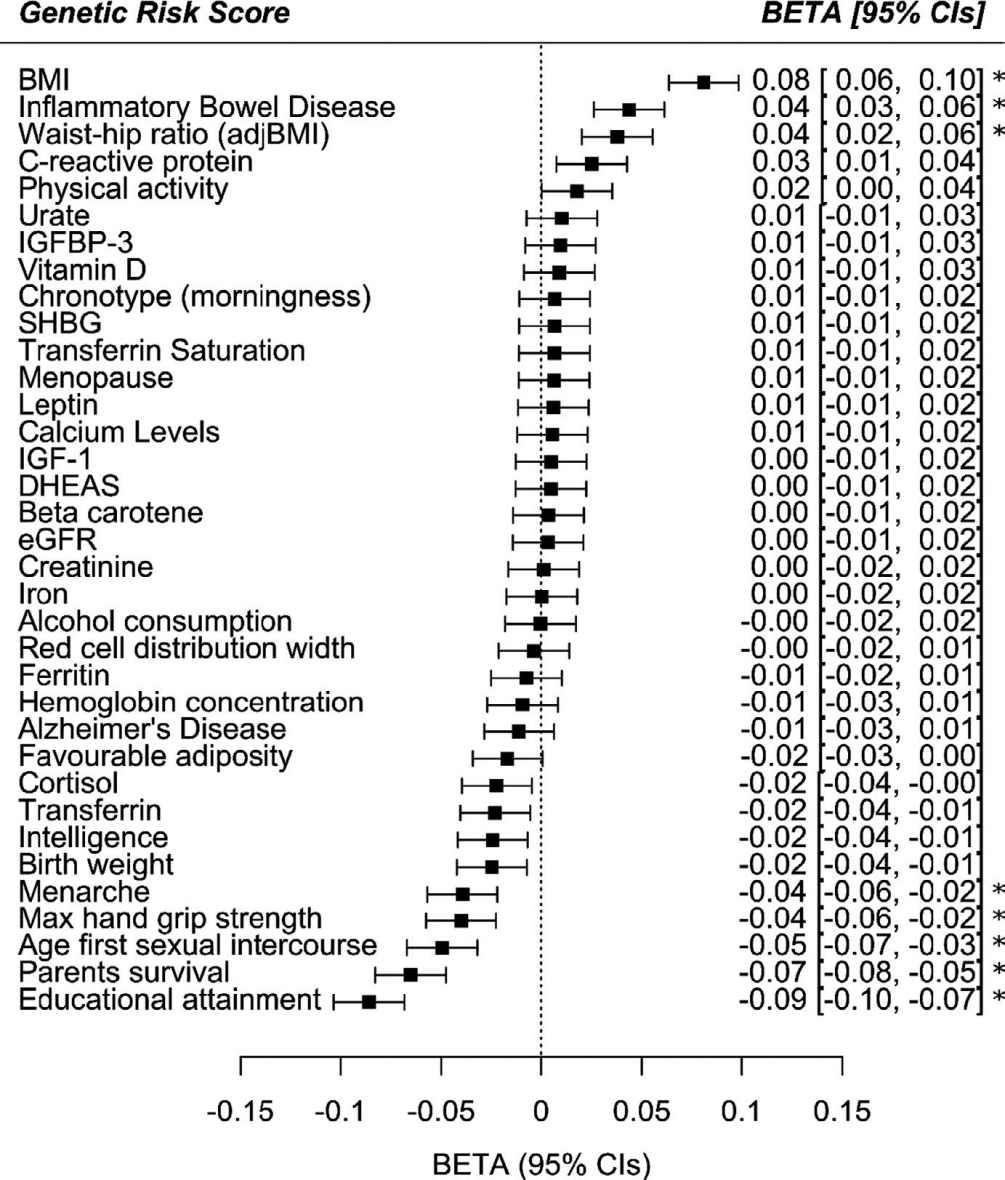
Genetic risk score associations with the frailty index in UK Biobank. Thirty‐five exposures, including lifestyle factors, clinical measures, circulating biomarkers and diseases, were assessed for their association with the Frailty Index by genetic risk score analysis in UK Biobank participants of European descent aged 60–70 years. Linear regression models included age, sex, assessment centre (22 categories), genotyping array (2 categories: Axiom or BiLEVE) and principal components of ancestry 1–10 as covariates. The betas represent the SD change in FI per SD increase in genetic predisposition to the exposure. Positive betas suggest increased frailty in individuals with greater genetic predisposition to the exposure, whereas negative betas represent a protective effect with increasing genetic predisposition. See Table S10 for details. * = significant *p*<0.0014 after Bonferroni correction for 35 tests. Abbreviations: BMI = body mass index; adjBMI = adjusted for BMI; IGFBP‐3 = insulin‐like growth factor‐binding protein 3; SHBG = sex hormone binding globulin; IGF‐1 = insulin‐like growth factor 1; DHEAS = Dehydroepiandrosterone sulphate; eGFR = estimated glomerular filtration rate; CIs = 95% confidence intervals

To identify potentially modifiable phenotypic traits that may contribute to the development of frailty over the life course, we conducted a series of MR analyses. First, we calculated genetic risk scores (GRS) in UK Biobank for 35 traits for which we could retrieve one or more SNPs robustly associated with the traits from pre‐existing GWAS results (Figure 2 and Table S10). We then tested the association of each GRS with the FI in UK Biobank. Using two‐sample MR methods, we further examined the top 8 results from GRS analyses that passed Bonferroni correction for 35 tests (*p* < 0.0014): educational attainment, BMI, waist‐to‐hip ratio, inflammatory bowel disease, grip strength, parental attained age (as a proxy for inherited propensity for longevity), age at menarche and the age at first sexual intercourse (a proxy for a combination of personality characteristics and related health behaviours). We used summary statistics from an GWAS of untransformed Frailty Index (total points, rather than quantile normalized) in UK Biobank only, meaning effect sizes can be interpreted on the actual FI scale.

**FIGURE 3:**
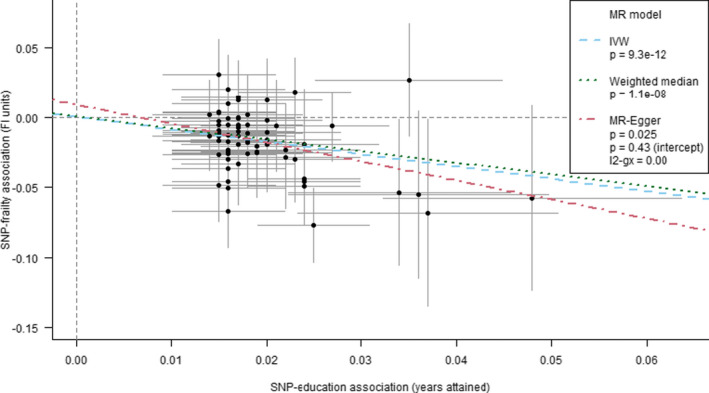
Mendelian randomization estimates for the effect of educational attainment on the frailty index in UK Biobank Points and error bars represent beta estimates and 95% confidence intervals for each SNP‐education / SNP‐FI association. The trend lines represent different methods for summarizing the estimates from individual SNPS—inverse variance weighting (IVW), weighted median and MR‐Egger. The weighted median and MR‐Egger estimates are less prone to bias from pleiotropy among the set of variants than IVW, given alternative assumptions hold. The MR‐Egger method includes a test of whether the trend's intercept differs from zero, which indicates whether there is an overall imbalance (directional) of pleiotropic effects: such bias was not identified in this education‐FI model

As trend lines in Figure 3 indicate, educational attainment was estimated to be a determinant of frailty by all model types: on average, a standard deviation increase (i.e. an additional 3.7 years) in education was predicted to lead to 13.6% lower frailty by the seventh decade of life in UK Biobank participants. There was no evidence to suggest that these results were influenced disproportionately by outlying variants, nor biased by directional pleiotropy (MR‐Egger intercept test *p* = 0.43), although the test for this may have had limited power. Higher BMI was associated with a higher risk of frailty, and later age of menarche was associated with a lower risk of frailty, although with more modest and inconclusive estimates from MR‐Egger and robust/penalized models (see Figures S3‐S9 for scatter plots and full MR results for the non‐education phenotypes tests). Model types also produced inconclusive or more divergent findings for grip strength, waist‐to‐hip ratio, inflammatory bowel disease, parental attained age and age at first sexual intercourse, at least partly reflecting the use of less informative sets of genetic instruments for analyses of several of these traits.

## 3 DISCUSSION

In this GWAS, we identified 14 genetic loci associated with frailty (as measured by the FI) in 164,610 UK community‐based individuals aged 60 to 70 years of European ancestry, with meta‐analysis of data from similarly aged Swedish individuals in TwinGene. We found suggestive evidence for others but more evidence is required to confirm these. Many of these loci have previously been associated with traits such as body mass index, cardiovascular disease, smoking initiation, HLA proteins, depression and neuroticism, suggesting roles for known disease risk factors and mental health in the development of frailty, with particular emphasis on genes expressed in the frontal cortex and hippocampus in the tissue expression analysis.

Our SNP‐based heritability estimate of 11% for the FI was slightly lower than previous family‐ or twin‐based estimates between 30 and 45% (Livshits et al., 2018) (Young et al., 2016) (Kim et al., 2013). This is expected as this SNP‐based estimate does not include rare, structural, non‐additive, or non‐autosomal genetic influences. Although we observed high genomic inflation *(Lambda GC* = *1*.*32)*, this is plausibly due to the FI being highly polygenic, with many genetic variants across the genome influencing an individual's frailty status (Bulik‐Sullivan et al., 2015).

The majority of FI‐associated loci have previously been identified for traits that have widespread health consequences, such as BMI, smoking initiation, depression, or neuroticism. The results include variants in the major histocompatibility complex region of chromosome six, containing many *HLA* (Human Leukocyte Antigen) genes. HLAs are cell‐surface proteins crucial for regulating immune function, which is known to decline with age (Simon et al., 2015). Genetic variants affecting the efficiency of immune function at older ages could have profound consequences for morbidity: variants in this region are robustly implicated in many chronic diseases and traits (Buniello et al., 2019), and also weakness in older people (sarcopenia) (Jones et al., 2019).

In our analysis of blood‐based DNA methylation associated with the FI, the only confirmed mQTL CpG (cg20614157) association was in a promotor for the *TNXB*/*TNXA*/*STK19* [Tenascin XB/putative Tenascin XA/Serine/threonine‐protein kinase 19] gene cluster, located in the *HLA* region. The association of a marker of potential functional relevance with the FI increases the likelihood that one or more of the genes in this cluster are of causal relevance for frailty risk, rather than others in the wider region. We found that high methylation level in cg20614157 was associated with higher frailty; a previous study has found that high methylation of the CpG site is associated with low expression of *TNXB* in breast and skin cancer tissues (Broad Institute of MIT & Harvard, 2016). *TNXB* is an extracellular matrix protein thought to be involved in wound healing and collagen function. Further research is needed to examine the potential role of the methylation and expression of *TNXB*, or other genes in this region, with frailty.

In tissue enrichment analysis, genes expressed in several brain regions are significantly downregulated by genetic variants associated with FI. Regions include the frontal cortex and hippocampus, the latter especially has been linked to dementia (Moodley & Chan, 2014). The main genes associated with several of the lead SNPs are linked directly to neuronal function, including *ANK3* (Ankyrin 3) which is a scaffold protein involved in neurotransmission with links to bipolar disorder and Alzheimer's disease (Hori et al., 2014) (Franceschi et al., 2018), *NLGN1* (Neuroligin 1), a synapse cell‐surface protein implicated in autism spectrum disorder (Bottos et al., 2011), *HTT* (Huntingtin), linked to Huntington's disease and may have a role in vesicle formation in autophagy, and *SYT14* (Synaptotagmin 14), which mediates membrane trafficking in synaptic transmission, and mutations in this gene cause spinocerebellar ataxia(Doi et al., 2011). These results are consistent with FI‐defined frailty having a neurological basis, as has been suggested by a previous study showing that frailty and chronic widespread pain have shared neurological pathways (Livshits et al., 2018). Pain questionnaire responses constituted a notable proportion of the FI items in UK Biobank (9 of 49; 18.4%), which will have influenced these findings. However, the relationship of these genetic risk loci with frailty was also present in the TwinGene meta‐analysis (where equivalent data were available), which included fewer items related to pain perception (2 of 44; 4.5%).

A previous FI GWAS (Mekli et al., 2018) identified two variants (rs6765037 and rs7134291), but neither were associated with FI in UK Biobank, which was more than ten times larger than this previous study. Surprisingly, loci implicated in parental lifespan (Wright et al., 2019) (RHJ Timmers et al., 2019) or key ageing hallmarks (Pilling et al., 2016) were not associated with the FI at genome‐wide significance. This included genetic variants in *APOE*, 9p21.3 (*CDKN2A*/*B*), *TERT* and *FOXO3A*. The FI is strongly associated with morbidity and mortality epidemiologically, but it may be limited as a tool for identifying specific ageing pathways, given the broad definition that includes many diverse diseases and characteristics.

In Mendelian randomization analyses, we tested whether long‐term effects of specific physiological, behavioural or lifestyle factors may affect FI scores. The most prominent findings indicated that predispositions to higher educational attainment and lower BMI were related to decreased frailty. Higher BMI directly affects cardiovascular health and subsequent disease risk, in addition to possible indirect effects by reducing socioeconomic status due to stigma (especially in women) (Tyrrell et al., 2016), with subsequent effects on frailty. This also supports a recent study which provided evidence against the notion of an obesity paradox in older people (Bowman et al., 2017).

To our knowledge, this is the largest genetic study of the frailty index to date. However, this study is limited to participants of European ancestry so results may not be generalizable to other populations. UK Biobank volunteers tend to be healthier and less socioeconomically deprived at baseline than the general UK population (Fry et al., 2017), perhaps reducing power to detect variants associated with the FI. The FI was also based on self‐reported baseline data so is open to the possibility of misclassification bias, but the FI has previously been validated in UK Biobank and shown to be strongly predictive of all‐cause mortality (Williams et al., 2019). Sample sizes in TwinGene and SATSA are also relatively small so power for the replication and CpG‐FI analyses may have been limited. Although we show that the FI definitions are comparable between UK Biobank and TwinGene, differences in genetic background could also have contributed to the limited overlap in results.

In conclusion, frailty is influenced by a large number of genetic determinants linked to well‐known disease risk factors such as BMI, cardiovascular disease, smoking and HLA proteins. Frailty is also influenced by genetic determinants for depression and neuroticism, suggesting a role for mental health, which may be underpinned by pathways linked to brain function. Future research is required to replicate our results in other cohorts, testing for consistency of genetic determinants when frailty has been measured differently. In particular, a comparable GWAS of the frailty phenotype, based on physical components of health (e.g. exhaustion, weakness) rather than comorbidities, would be likely to yield further risk loci specifically for these components of frailty.

## 4 EXPERIMENTAL PROCEDURES

### 4.1 UK Biobank

UK Biobank recruited 502,642 community volunteers, aged 37 to 73 years old, from 22 assessment centres across England, Scotland and Wales between 2006 and 2010 (Sudlow et al., 2015). At baseline, participants provided self‐reported information on demographics, lifestyle and disease history via questionnaire and underwent physiological measurements, including providing a blood sample for genetics data. We included participants aged 60 to 70 years old at baseline of European descent, with genetics data (*n* = 201,062) and available data to generate the frailty index (*n* = 164,610) (see Frailty Index section below for details). UK Biobank has ethical approval from the North West Multi‐Centre Research Ethics Committee.

### 4.2 TwinGene

TwinGene data collection took place between 2004 and 2008, when the older participants of the Screening Across the Lifespan Twin (SALT) study were invited to donate blood for molecular and genetic analyses. Both same‐ and opposite‐sex twins were included. Ascertainment procedures for SALT (Lichtenstein et al., 2002) and TwinGene (Magnusson et al., 2013) have been previously described. This study was based on the sample that had both genetic and FI data available (*n* = 10,616). All participants have given their informed consent. The TwinGene study was approved by the Regional Ethics Review Board, Stockholm.

### 4.3 Swedish Adoption/Twin Study of Aging (SATSA)

SATSA is a longitudinal study in gerontological genetics, with sampling of participants drawn from the Swedish Twin Registry. SATSA was initiated in 1984 and ended in 2014, and it comprises of nine questionnaire and ten in‐person testing (IPT) waves. The participants are same‐sex twins, of which some twin pairs have been reared together and some separated before age 11 and reared apart. Ascertainment procedures for SATSA have been described previously (Finkel & Pedersen, 2004). This study made use of IPT data, and participants’ first available measurement on FI and DNA methylation were included (*n* = 368). All participants have provided informed consent. The SATSA study was approved by the Regional Ethics Review Board in Stockholm.

### 4.4 Frailty Index (FI)

We used an FI based on the accumulation of deficits model (Searle et al., 2008), as validated in UK Biobank previously (Williams et al., 2019). The FI was derived using 49 self‐reported baseline data variables in UK Biobank. Variables were based on a variety of physiological and mental health domains, and included symptoms, disabilities and diagnosed diseases, which were self‐reported by participants at baseline (see Table S11 for details of the FI components included and the proportion of individuals scoring one for each component). The FI was generated using a complete‐case sample with information on all 49 individual components (*n* = 164,610) and presented as a proportion of the sum of all deficits. The FI was quantile normalized (i.e. transformed into a normal distribution) prior to the genome‐wide association study (due to the skew of the untransformed trait) and sensitivity analyses using the non‐transformed trait were performed.

Construction of the FIs for TwinGene and SATSA have been previously described and validated for their ability to predict mortality (Li et al., 2019) (Raymond et al., 2020). Briefly, both the TwinGene FI and SATSA FI were constructed using self‐reported questionnaire data (for TwinGene, the data collected in SALT were used), and they cover a variety of different health domains. The TwinGene/SALT FI consists of 44 deficits (Table S12), and the SATSA FI consists of 42 deficits. Prior to the analyses, the FIs were quantile normalized.

We compared the FIs used in UK Biobank and TwinGene. Of the 49 items used in UK Biobank, 29 of these have approximate items in TwinGene (see Table S13 for details of overlap). The subset of 29 items in UK Biobank were well correlated with the full 49 items (*r* ^2^ = 0.85, *p *< 0.0001).

### 4.5 GWAS Meta‐analysis of the Frailty Index

We used data from UK Biobank v3 genotyping release, described in detail previously (Bycroft et al., 2018). In brief, 488,377 participants were successfully genotyped using custom Affymetrix microarrays for ~820,000 variants. Imputation was performed using 1000 Genomes and the Haplotype Reference Consortium (HRC) reference panels, yielding ~93 million variants in 487,442 participants. Participants were excluded if the UK Biobank team flagged them as either having unusually high heterozygosity or missing genotype calls (*n* = 968) (Bycroft et al., 2018). Our analysis was restricted to those of European descent (*n* = 451,367, method previously published (Thompson et al., 2019)), aged 60 to 70 with complete FI data (*n* = 164,610). We analysed 16,446,667 autosomal variants with minor allele frequency (MAF) >0.1%, Hardy–Weinberg p‐value >1 × 10^−9^ and imputation quality >0.3. BOLT‐LMM (v2.3.2) software used for the GWAS itself (Loh et al., 2015), which uses linear mixed‐effects modelling to account for genetic relatedness and confounding by ancestry. Models included age, sex, assessment centre (22 categories) and genotyping array (two categories: Axiom or BiLEVE) as covariates.

Genotyping of the TwinGene was performed using the Illumina OmniExpress platform and has been previously described (Magnusson et al., 2013). The present study made use of data imputed using the Haplotype Reference Consortium (HRC) reference panel, and the samples were processed using the Ricopili (Broad Institute RICOPILI) pipeline for quality control (QC). 29 (0.3%) samples failed sample QC due to either of the following: per‐sample call rate <0.98; excessive heterozygosity (FHET outside +/− 0.20); and sex mismatch. 41,972 out of 731,442 (5.7%) failed SNP QC due to either of the following: per‐SNP call rate <0.98; invariant; and Hardy–Weinberg disequilibrium (*p* < 1e‐6). By projecting the first two principal components (PCs) of the study samples to the reference panel of 1000 Genome global population, we identified 6 (0.05%) samples as non‐European ancestral outliers, whose first two PCs exceeded 6 SDs from the mean values of the European samples in the reference population. We reran QC after the removal of the ancestral outliers and further dropped 1 sample and 4 SNPs that failed the second QC. We then used the Sanger imputation service [https://imputation.sanger.ac.uk/] to impute the post‐QC genotype data to the reference panel of Haplotype Reference Consortium data (HRC1.1) (McCarthy et al., 2016). EAGLE2 (Loh et al., 2016) and IMPUTE2 (Howie et al., 2009) were used for prephasing and imputing, respectively. 40 million SNPs were available after the HRC imputation. SNPs with MAF<0.1% were excluded from further analysis. Associations were tested using a linear regression under an additive assumption for genotype dosages and adjusting for age, sex and ten first principal components. Clustering of the data in twin pairs was accounted for by using cluster robust standard errors. Stata version 15.1 (College Station, TX: StataCorp LP) was used in the analysis.

Fixed‐effects meta‐analysis was performed on 7,666,890 genetic variants that passed QC in both cohorts. METAL software was used to perform the analysis, with genomic control for population structure (Willer et al., 2010).

We used Linkage Disequilibrium Score Regression (LDSR, v1.0.0) to estimate the level of bias (e.g. from population stratification and cryptic heritability) in the GWAS, and the heritability of the Frailty Index (Bulik‐Sullivan et al., 2015).

### 4.6 Gene Ontology Pathways & QTL analyses

See Supplementary Methods for details.

### 4.7 Mendelian Randomization (MR)

MR is the application of genetic variation to infer whether phenotypic traits or exposures affect diseases or health‐related outcomes (Lawlor et al., 2008). We investigated a range of exposures for which genetic determinants (typically SNPs) have been identified in previous GWAS. In total, 35 exposure‐frailty associations were modelled in UK Biobank. Exposures included lifestyle factors, clinical measures, circulating biomarkers and diseases. All traits are listed in Table S10, with references for the GWAS from which sets of instrumenting variants were sourced. We used genetic risk scores (GRS) for each trait in initial MR models, and followed up several of the lead findings with sensitivity analyses to detect and account for bias by pleiotropy among genetic instruments (described in the Supplementary Methods).

### 4.8 Methylation—FI association analysis

To identify whether risk variants could be related to frailty via DNA methylation differences, we assessed whether the FI‐associated genetic variants harboured methylation quantitative trait loci (mQTL) and whether methylation levels in such loci demonstrated further associations with the FI in SATSA (see Supplementary Methods for further details).

## CONFLICTS OF INTEREST

No conflicts of interest to declare.

## AUTHOR CONTRIBUTIONS

JLA and LCP drafted the manuscript. JLA derived the FI in UK Biobank and performed descriptive statistics on the data. LCP performed GWAS and pathways analysis in UK Biobank. DMW performed QTL look‐ups and Mendelian randomization analysis in UK Biobank. YW performed genetic analysis in Twin Gene and JJ performed mQTL analyses. PKM and NLP were responsible of acquisition of the TwinGene genetic data. NLP coordinates the SATSA study, and together with SH, was responsible of acquisition of the SATSA methylation data. YL was responsible for imputing and pre‐processing the TwinGene genotype data. YW was responsible of pre‐processing the SATSA methylation data. All authors contributed to the design of study, data interpretation and revision of the manuscript.

## Supporting information

Supplementary Material

Table S1‐S13

## Data Availability

Data are available on application to the UK Biobank (Sudlow et al., 2015) (www.ukbiobank.ac.uk/register‐apply). Full GWAS summary statistics are freely available to download via the GWAS catalog (https://www.ebi.ac.uk/gwas/downloads/summary‐statistics; study accession GCST90020053) or directly from (https://doi.org/10.6084/m9.figshare.9204998).
