# Supplementary material for: A genome‐wide association study of the frailty index highlights brain pathways in ageing
Source: Aging Cell. 2021 Aug 25;20(9):e13459. doi: 10.1111/acel.13459 (PMC8441299; doi:10.1111/acel.13459)
Supplement: Supplementary file 1 — Supplementary Material [file ACEL-20-e13459-s001.docx]

**A Genome-Wide Association Study of the Frailty Index**

Atkins *et al.* 2021

**Supplementary Information**

[Supplementary Methods 3](#_Toc76734499)

[Gene Ontology Pathways Analysis 3](#_Toc76734500)

[GWAS Hits as Quantitative Trait Loci (QTLs) 3](#_Toc76734501)

[Methylation – FI Association Analyses 3](#_Toc76734502)

[Mendelian Randomization (MR) 3](#_Toc76734503)

[Supplementary Figures 5](#_Toc76734504)

[Supplementary Figure 1: QQ plot of Frailty Index GWAS 5](#_Toc76734505)

[Supplementary Figures 2A-N: LocusZoom plots for the 14 loci associated with FI in UK Biobank and TwinGene 6](#_Toc76734506)

[Supplementary Figure 2A: rs12739243 (1:210302043:T:C) 6](#_Toc76734507)

[Supplementary Figure 2B: rs4952693 (2:44151808:T:C) 6](#_Toc76734508)

[Supplementary Figure 2C: rs2071207 (3:50159844:T:C) 7](#_Toc76734509)

[Supplementary Figure 2D: rs583514 (3:173114167:T:C) 7](#_Toc76734510)

[Supplementary Figure 2E: rs82334 (4:3225371:A:C) 7](#_Toc76734511)

[Supplementary Figure 2F: rs1363103 (5:103917837:T:C) 8](#_Toc76734512)

[Supplementary Figure 2G: rs9275160 (6:32652620:A:G) 8](#_Toc76734513)

[Supplementary Figure 2H: rs2396766 (7:114318071:A:G) 8](#_Toc76734514)

[Supplementary Figure 2I: rs56299474 (8:21992804:A:C) 9](#_Toc76734515)

[Supplementary Figure 2J: rs4146140 (10:61885362:T:C) 9](#_Toc76734516)

[Supplementary Figure 2K: rs10891490 (11:112885527:T:C) 9](#_Toc76734517)

[Supplementary Figure 2L: rs3959554 (15:41443924:A:G) 10](#_Toc76734518)

[Supplementary Figure 2M: rs17612102 (15:52264094:T:C) 10](#_Toc76734519)

[Supplementary Figure 2N: rs8089807 (18:39322639:T:C) 10](#_Toc76734520)

[Supplementary Figure 3: Mendelian randomization: estimated effect of higher BMI on the frailty index in UK Biobank 11](#_Toc76734521)

[Supplementary Figure 4: Mendelian randomization: estimated effect of a higher liability to inflammatory bowel disease on the frailty index in UK Biobank 12](#_Toc76734522)

[Supplementary Figure 5: Mendelian randomization: estimated effect of a higher waist-to-hip ratio on the frailty index in UK Biobank 13](#_Toc76734523)

[Supplementary Figure 6: Mendelian randomization: estimated effect of liability for a higher age at menarche on the frailty index in UK Biobank 14](#_Toc76734524)

[Supplementary Figure 7: Mendelian randomization: estimated effect of higher grip strength on the frailty index in UK Biobank 15](#_Toc76734525)

[Supplementary Figure 8: Mendelian randomization: estimated effect of liability for a higher age at first sexual intercourse on the frailty index in UK Biobank 16](#_Toc76734526)

[Supplementary Figure 9: Mendelian randomization: estimated effect of higher parental survival on the frailty index in UK Biobank 17](#_Toc76734527)

[References 18](#_Toc76734528)

**Supplementary Tables** ………………………………………………………….See Excel spreadsheet

#

# Supplementary Methods

## Gene Ontology Pathways Analysis

MAGMA (Multi-marker Analysis of GenoMic Annotation) is a tool for gene set enrichment analysis designed to give a pathways-based summary of individual GWAS results (de Leeuw et al. 2015). We utilized the FUMA (Functional mapping and annotation of genetic associations) platform to perform this analysis with default options (Watanabe et al. 2017).

LD Score Regression applied to specifically expressed genes (LDSC-SEG) allows the identification of enriched tissue activity associated with GWAS results (Finucane et al. 2018). We applied LDSC-SEG (v1.0.0) to the FI GWAS summary statistics using the datasets `Multi_tissue_gene_expr` and `Multi_tissue_chromatin` provided by the authors. We applied Bonferroni correction for 703 tests (n gene expression = 205, n chromatin = 498) and accepted LDSC-SEG results p<7*10^-5^ as significant.

## GWAS Hits as Quantitative Trait Loci (QTLs)

For the 14 top GWAS hits, we downloaded SNP-QTL association statistics from previous proteomics, metabolomic, gene expression, and epigenetic GWAS datasets listed in a repository of genetic associations (Kamat et al. 2019), without using LD proxies for these SNPs and limiting associations to those with *p*-values under 5*10^-8^. Prominent sources of information included the INTERVAL study for proteomic QTLs (Sun et al. 2018), eQTLGen and GTEx datasets for expression QTLs (Võsa et al. 2018)(Ardlie et al. 2015), and ARIES, BIOS and BLUEPRINT studies for epigenetic QTLs (Gaunt et al. 2016)(Bonder et al. 2017)(Chen et al. 2016).

## Methylation – FI Association Analyses

Procedures for acquisition and processing of the SATSA genotype (Illumina PsychChip) and DNA methylation data (Illumina 450k chip) have been previously described (Wang et al. 2018). In this study, we sought to analyse if those 2,007 genetic variants that were significantly associated with the FI in the UK Biobank and TwinGene were among the *cis*-mQTL previously identified in SATSA (Wang et al. 2018). The *cis*-mQTL are genetic variants that are associated with methylation levels in nearby CpG sites (a distance <1 million base pairs between markers used in SATSA), hence representing a form of genetic regulation on DNA methylation. In SATSA, ~1.41 million associations between 6.5 million SNPs and 390,894 CpGs across the genome have been identified as statistically significant when using a Bonferroni-corrected threshold P=2.5x10^-11^ (Wang et al. 2018). After identifying those FI-associated SNPs in the UK Biobank/TwinGene GWAS meta-analysis that were among the *cis*-mQTL in SATSA, we further analysed whether the methylation levels in the *cis*-mQTL CpG sites were associated with the FI in SATSA. First available measurement of the FI was used, and for methylation data, we used the beta values (ranging from 0 to 1). The analysis was performed using a linear regression adjusting for age and sex and correcting for the standard errors due to clustering of the data in twin pairs. The analysis was performed using R version 3.4.1 and the standard errors were corrected using the"*multiwayvcov*" package.

## Mendelian Randomization (MR)

MR analyses were conducted in two stages. First, we assessed whether frailty measures were associated with genetic risk scores (GRS) for each trait in UK Biobank – weighted sums of alleles associated with a unit increase in a trait or disease risk. The GRS were entered as independent variables in each model, with covariates for age, sex, UKB baseline assessment centre attended, array type and the first ten principal components. Unlike main GWAS models, we calculated SNP-FI association statistics with the FI in its original scale, so that MR estimates are more easily interpreted as FI unit differences in frailty per long-term exposure to a unit increase in each trait. Associations of the GRS with a binary variable for frailty risk (an FI value ≥0.21 vs. lower values) were also assessed in logistic regression models, using the same covariates.

Second, we conducted additional MR models to further test the robustness of the top GRS results. Exposures eligible to model in the second stage were all those proxied by three or more SNPs, and with *p-*values under 0.0014 in GRS associations with frailty (an α threshold of 0.05 after Bonferroni correction for 35 independent tests). Several of the top exposure results were omitted from stage two follow-up because they were directly related to the FI composition – e.g. GRS results for cardiovascular risk factors measures, because cardio-metabolic traits constituted approximately ~20% of items used to compose the FI.

The additional MR models employed a two-sample MR design, where instrumental variable (IV) methods combine SNP-exposure and SNP-frailty estimates from separate GWAS to estimate the long-term effects of exposure variation on outcomes. Individual IV estimates from each SNP were combined using several meta-analysis methods for comparison: i) inverse variance weighting; ii) weighted median; iii) MR-Egger regression and its accompanying metrics to test for genetic pleiotropy and bias from measurement error in SNP-exposure associations (Burgess et al. 2017). These methods provide different model assumptions and degrees of adjustment for potential bias from pleiotropy, which can violate key assumptions of MR models. In sensitivity analyses, we also repeated analyses with ‘robust’ and ‘penalised’ versions of these estimators, which are less sensitive to outlying estimates, i.e. from SNPs which might be the most pleiotropic (Bowden et al. 2016). MR analyses made use of the ‘*MendelianRandomization’* R package (Yavorska & Burgess 2017).

# Supplementary Figures

## Supplementary Figure 1: QQ plot of Frailty Index GWAS


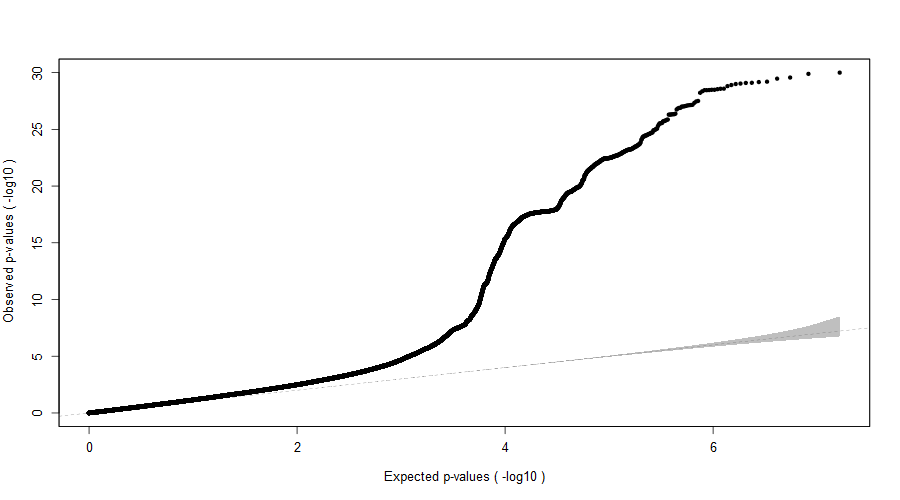


QQ plot of GWAS of Frailty Index (quantile normalized) against 7,666,890 autosomal variants in meta-analysis of UK Biobank and TwinGene data. We used LD Score Regression (LDSC, v1.0.0) to estimate the level of bias (e.g. from population stratification and cryptic heritability) in the GWAS, and the heritability of the Frailty Index (Bulik-Sullivan et al. 2015). The Lambda GC (genomic control) value was high (1.32), however the LD Score Regression intercept was close to the null (1.02: SE 0.009), indicating that the inflation in test statistics is due to polygenicity (many variants with small effects on frailty) rather than bias due to population stratification.

## Supplementary Figures 2A-N: LocusZoom plots for the 14 loci associated with FI in UK Biobank and TwinGene

The LocusZoom online tool (http://locuszoom.org) was used to plot the regions around the 14 loci significantly (p<5*10-8) associated with Frailty Index. The results can be explored using the below link.

<https://my.locuszoom.org/gwas/100819/?token=01cb3aafc1764e40a96519c12b7b186c>

## Supplementary Figure 2A: rs12739243 (1:210302043:T:C)


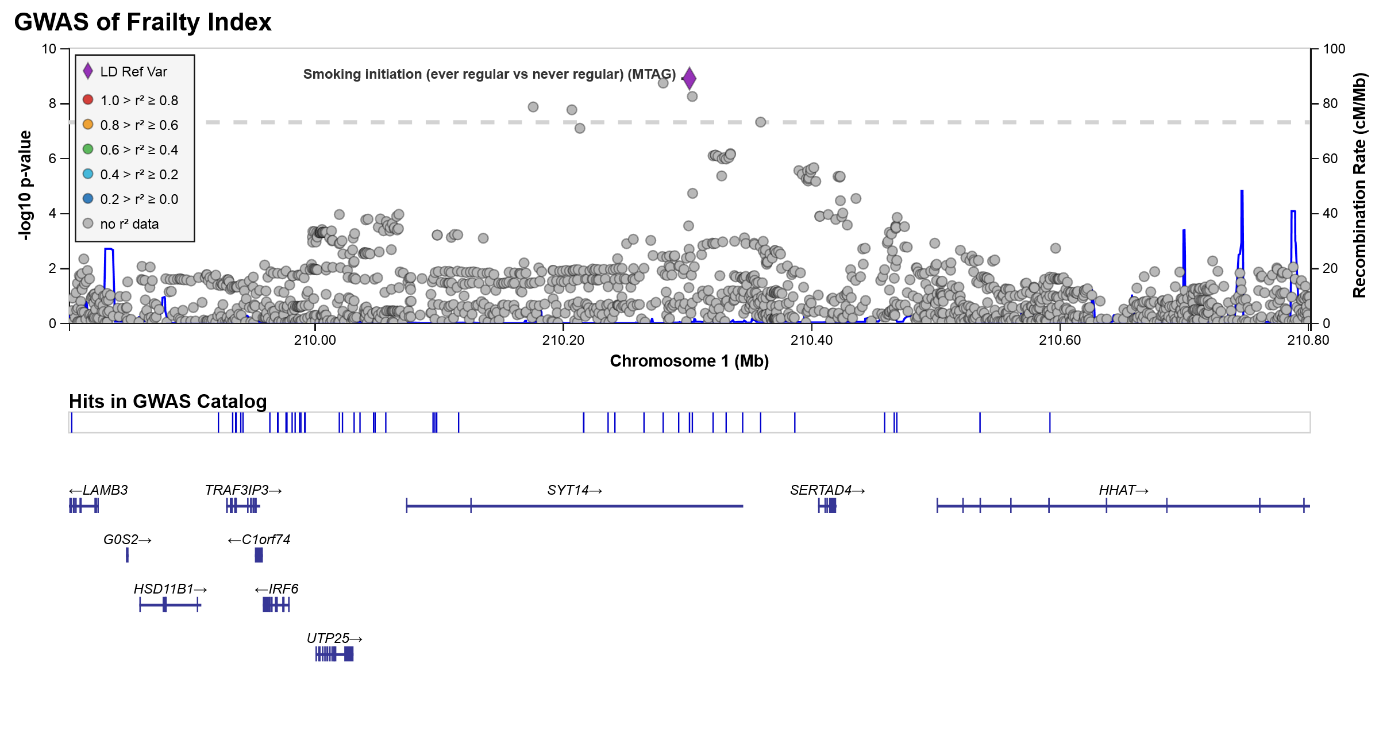


## Supplementary Figure 2B: rs4952693 (2:44151808:T:C)


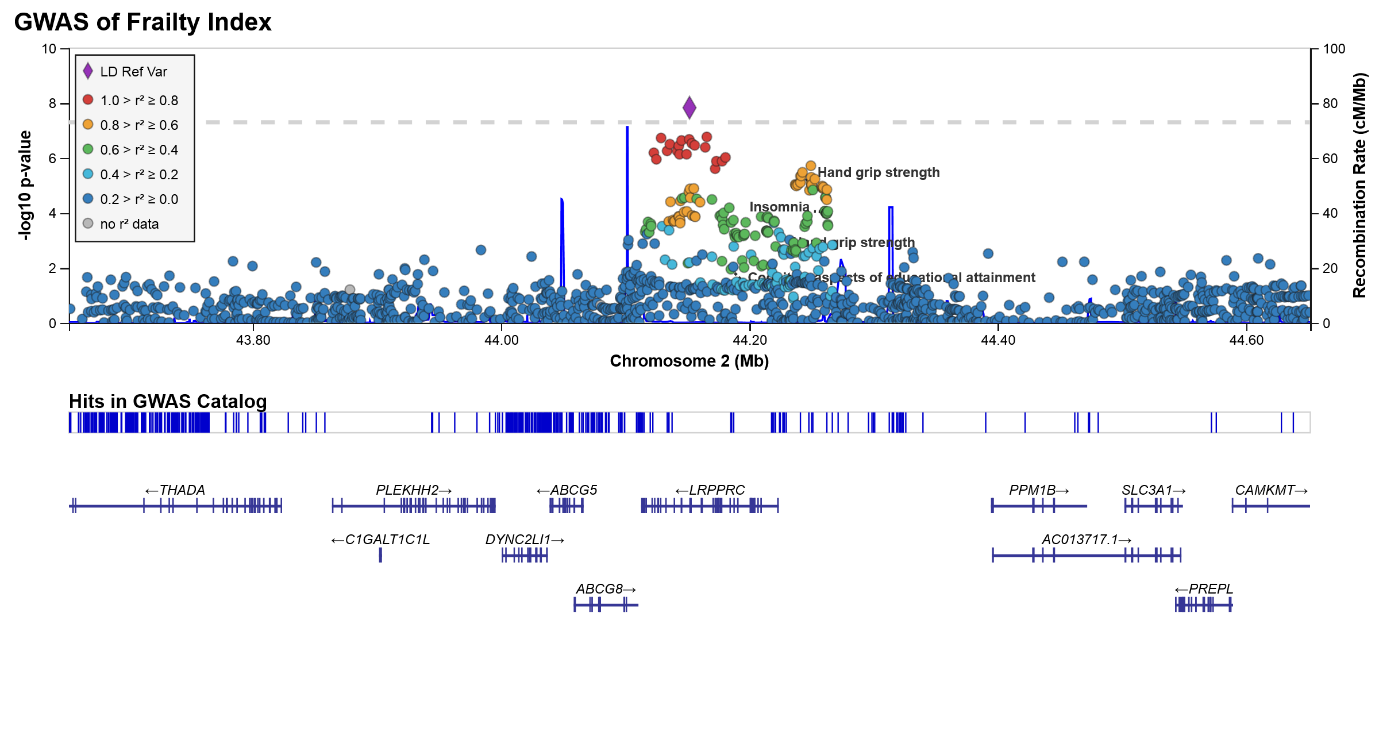


## Supplementary Figure 2C: rs2071207 (3:50159844:T:C)


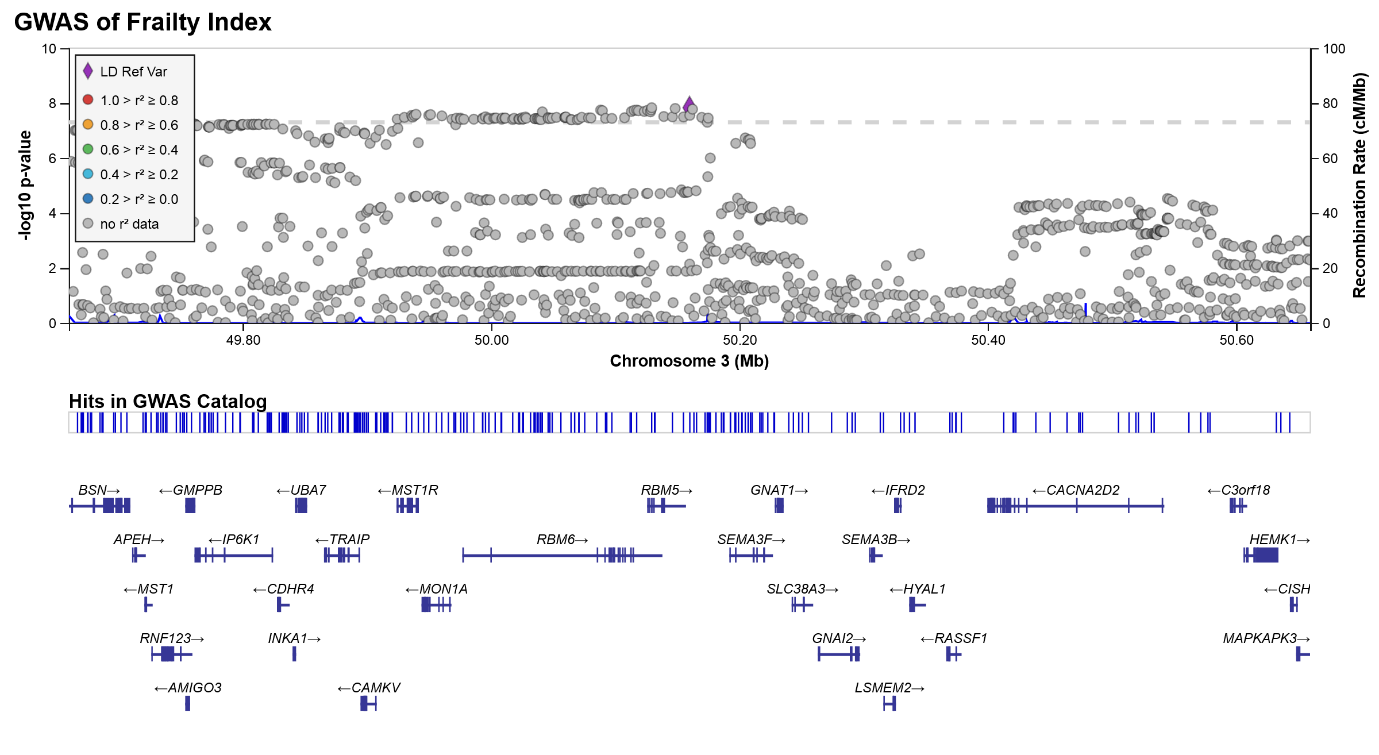


## Supplementary Figure 2D: rs583514 (3:173114167:T:C)


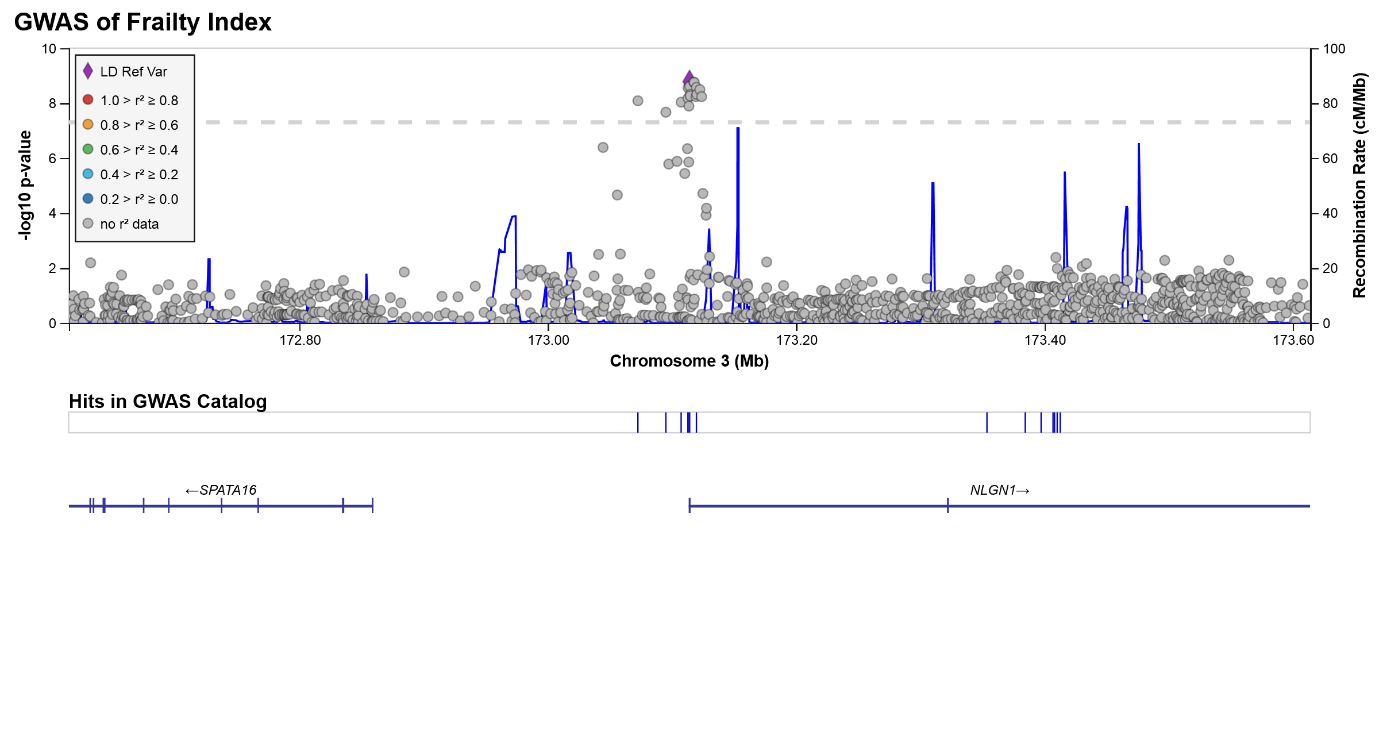


## Supplementary Figure 2E: rs82334 (4:3225371:A:C)


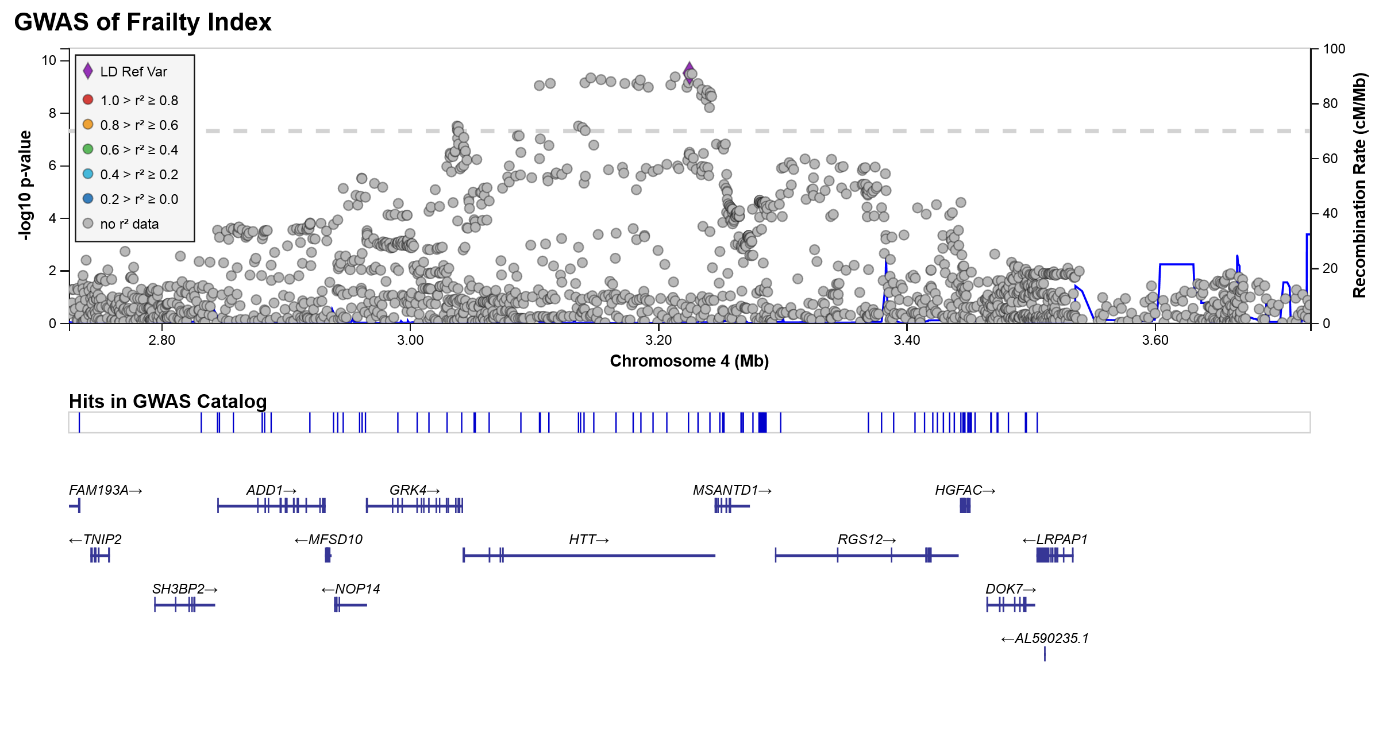


## Supplementary Figure 2F: rs1363103 (5:103917837:T:C)


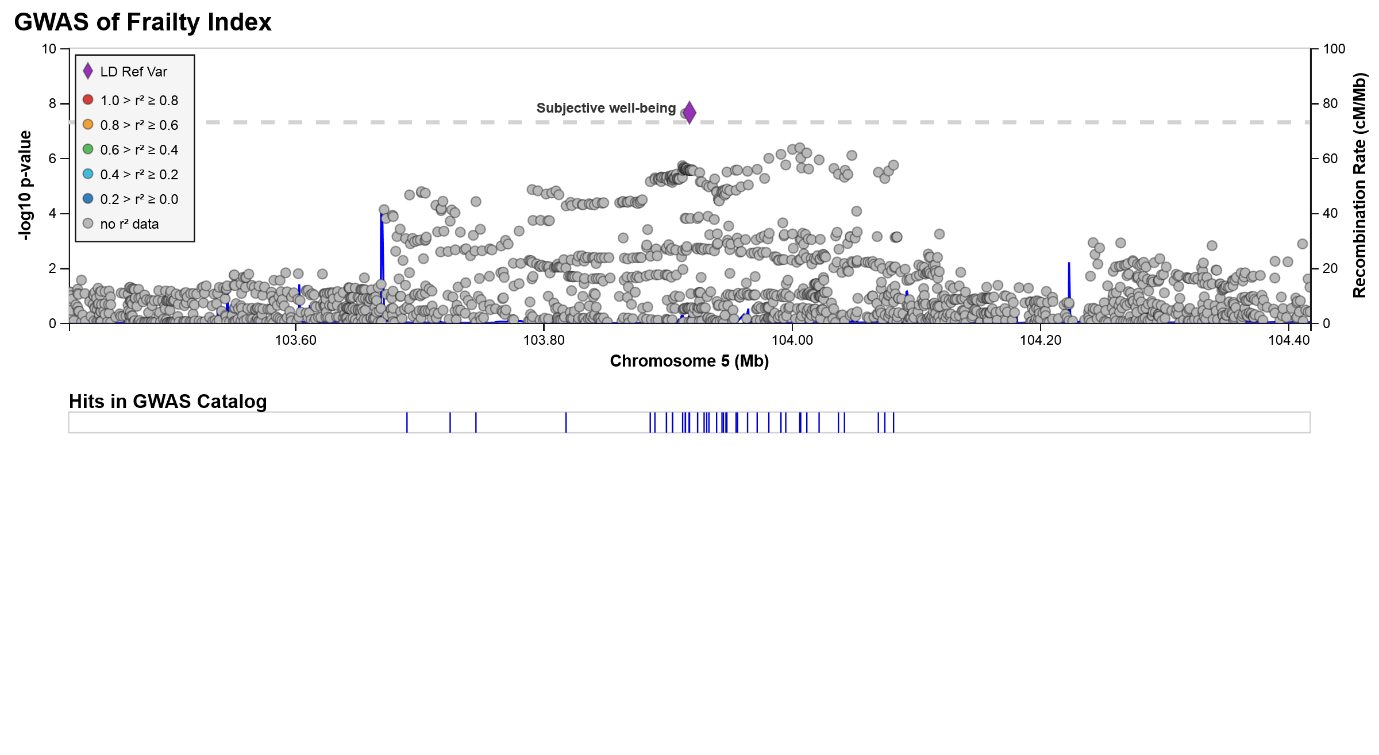


## Supplementary Figure 2G: rs9275160 (6:32652620:A:G)


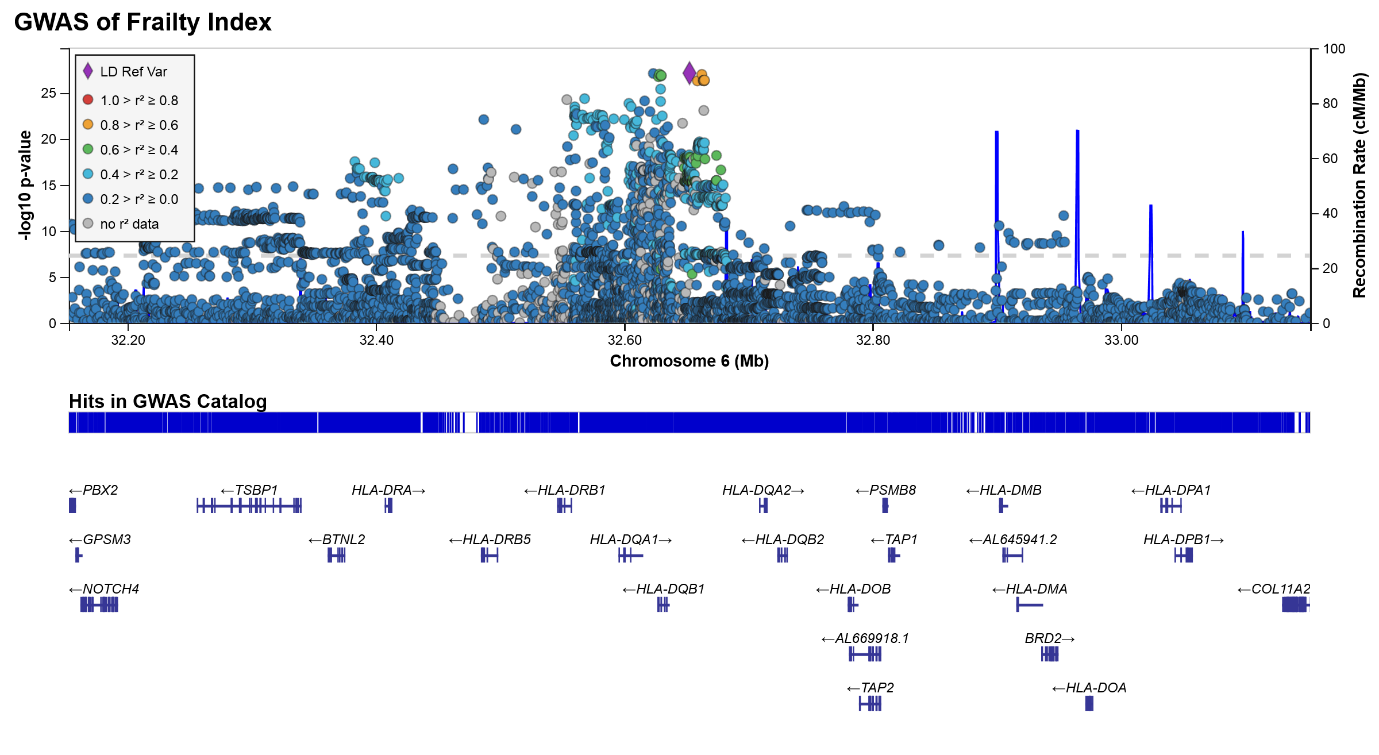


## Supplementary Figure 2H: rs2396766 (7:114318071:A:G)


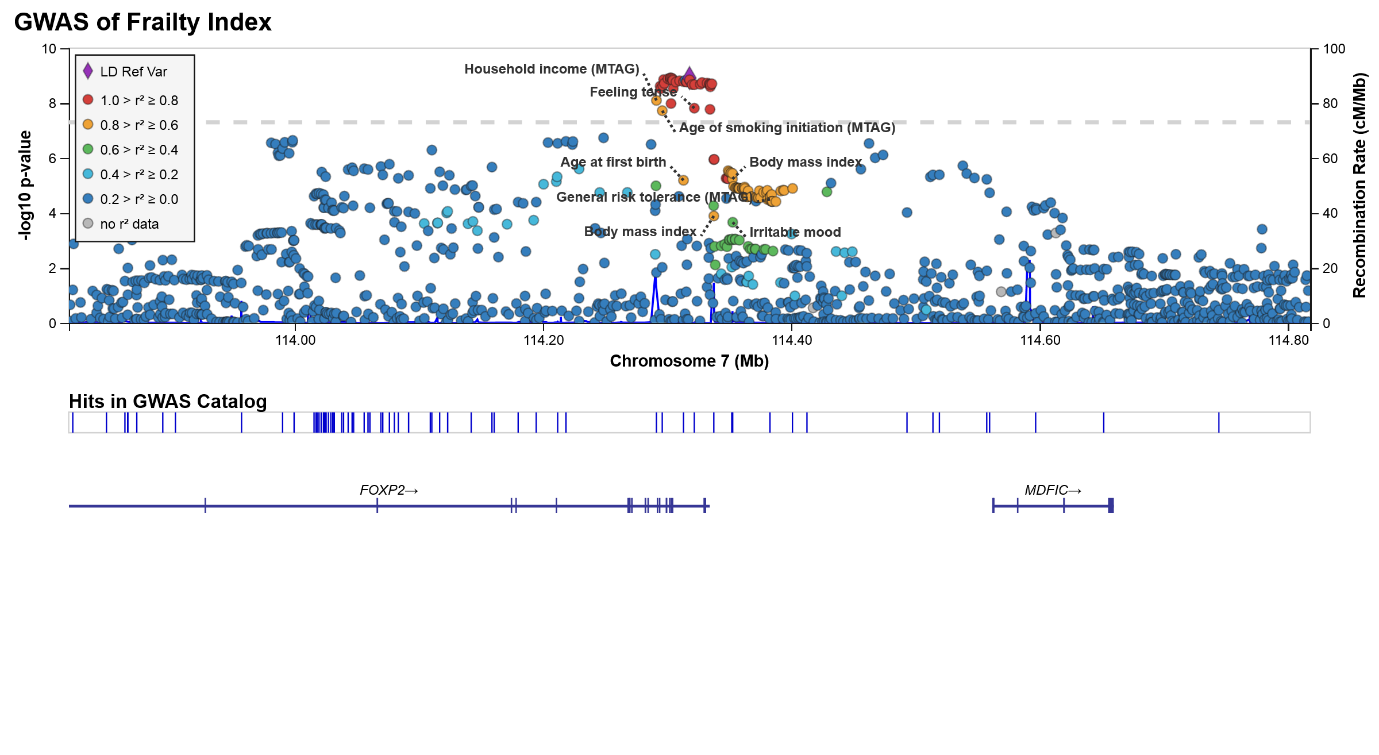


## Supplementary Figure 2I: rs56299474 (8:21992804:A:C)


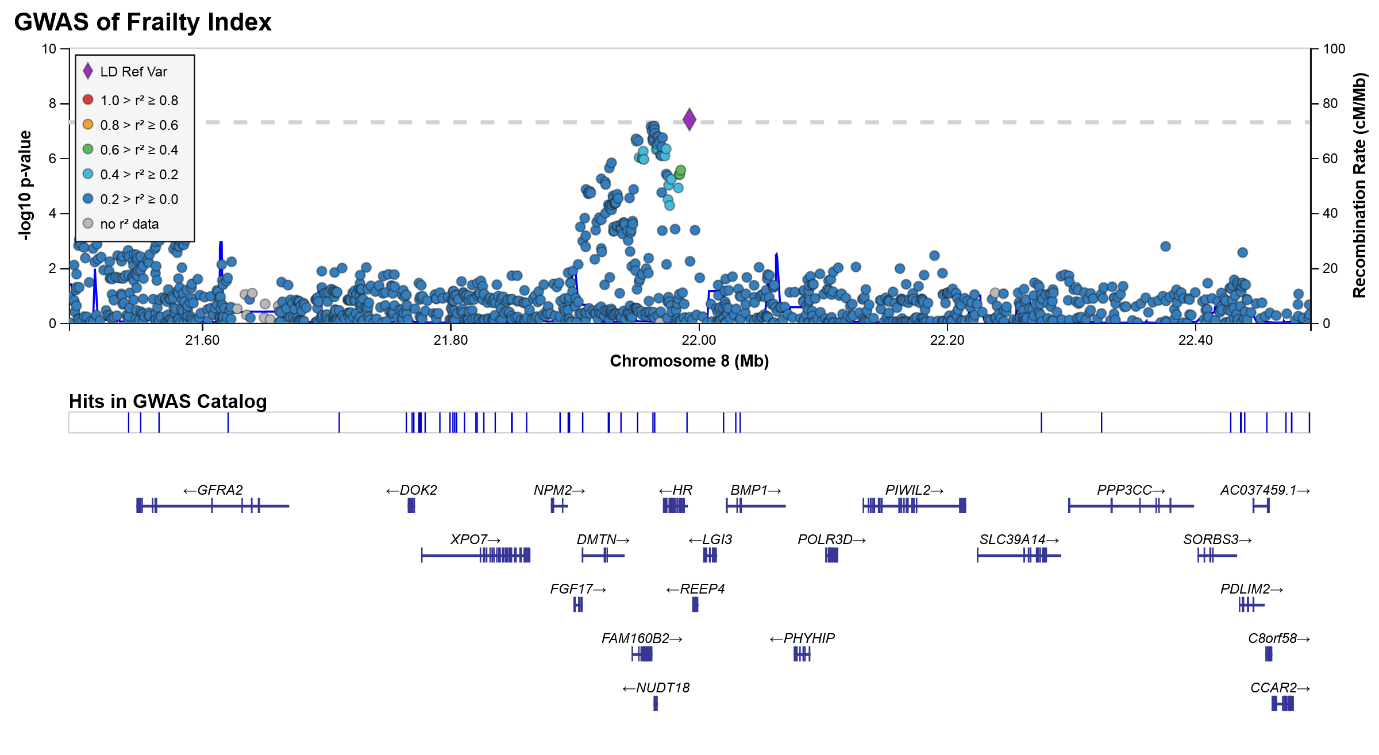


## Supplementary Figure 2J: rs4146140 (10:61885362:T:C)


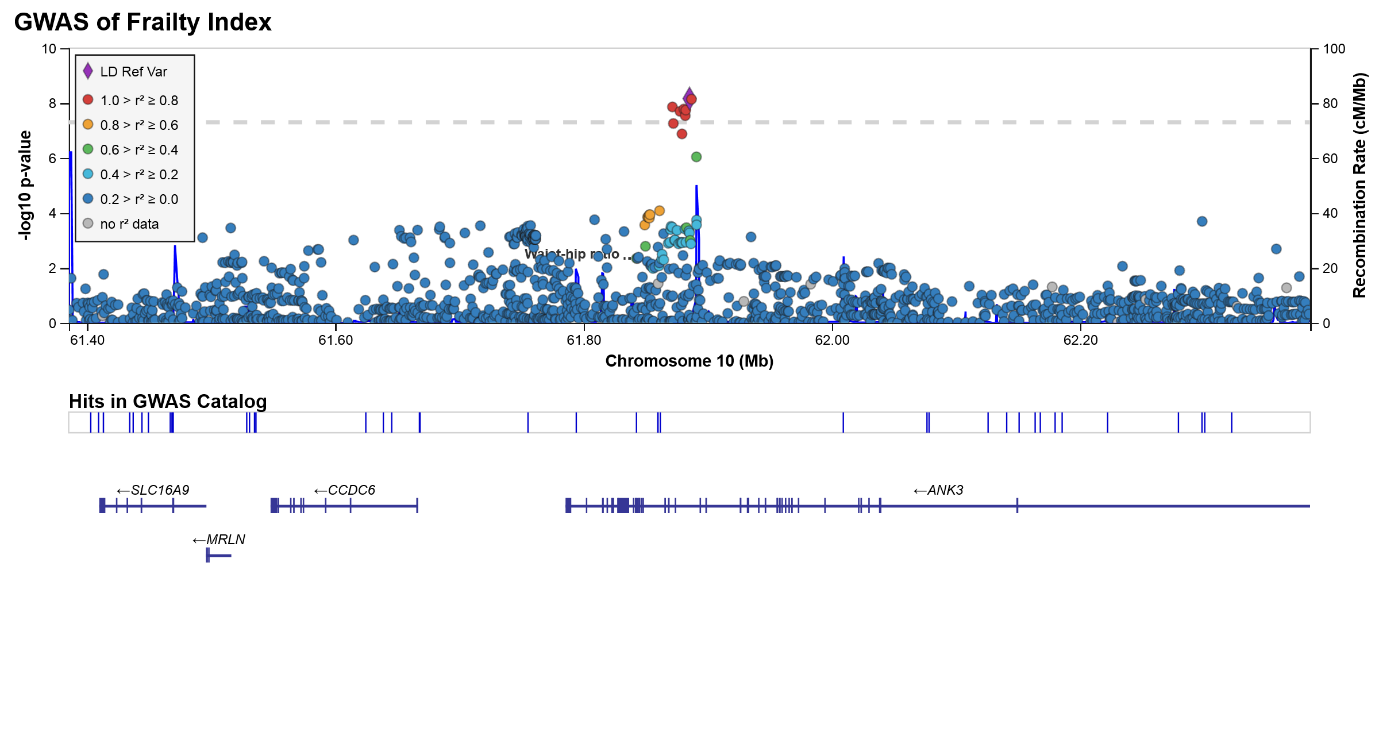


## Supplementary Figure 2K: rs10891490 (11:112885527:T:C)


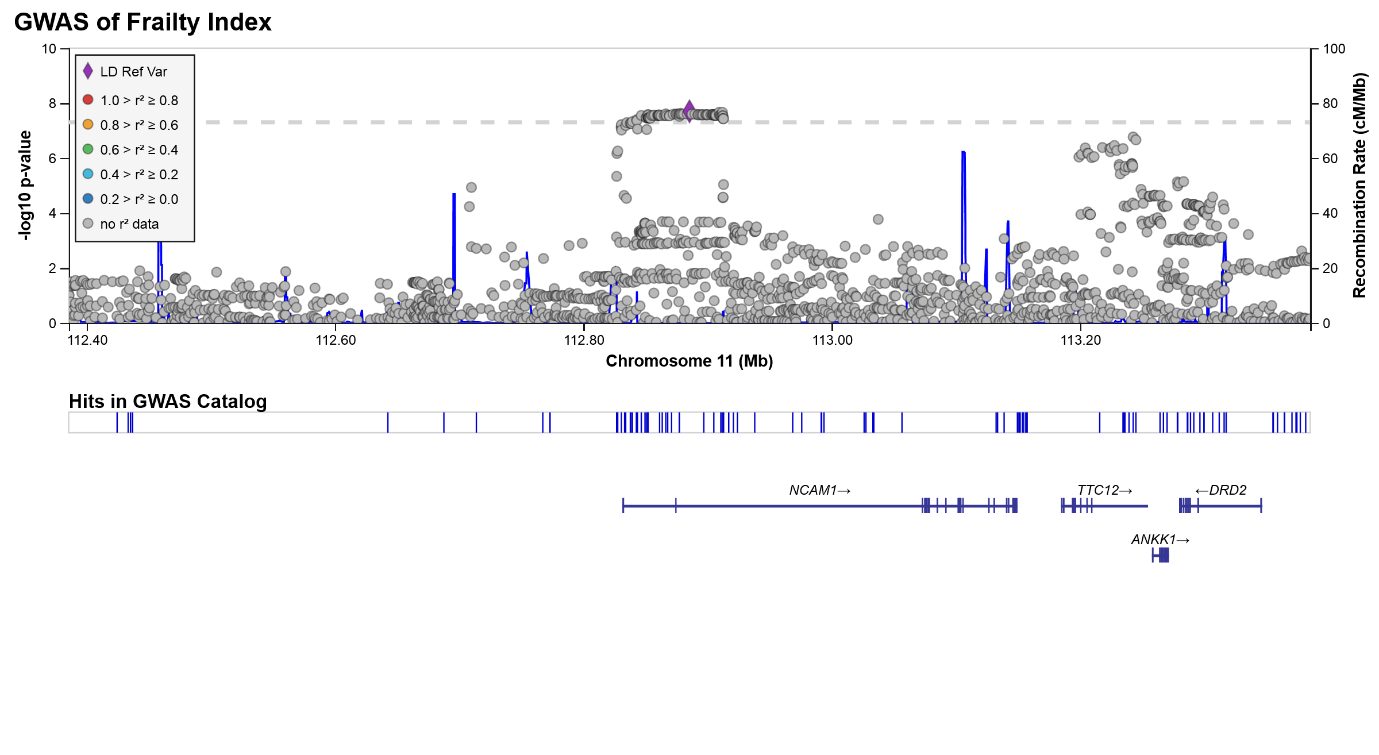


## Supplementary Figure 2L: rs3959554 (15:41443924:A:G)


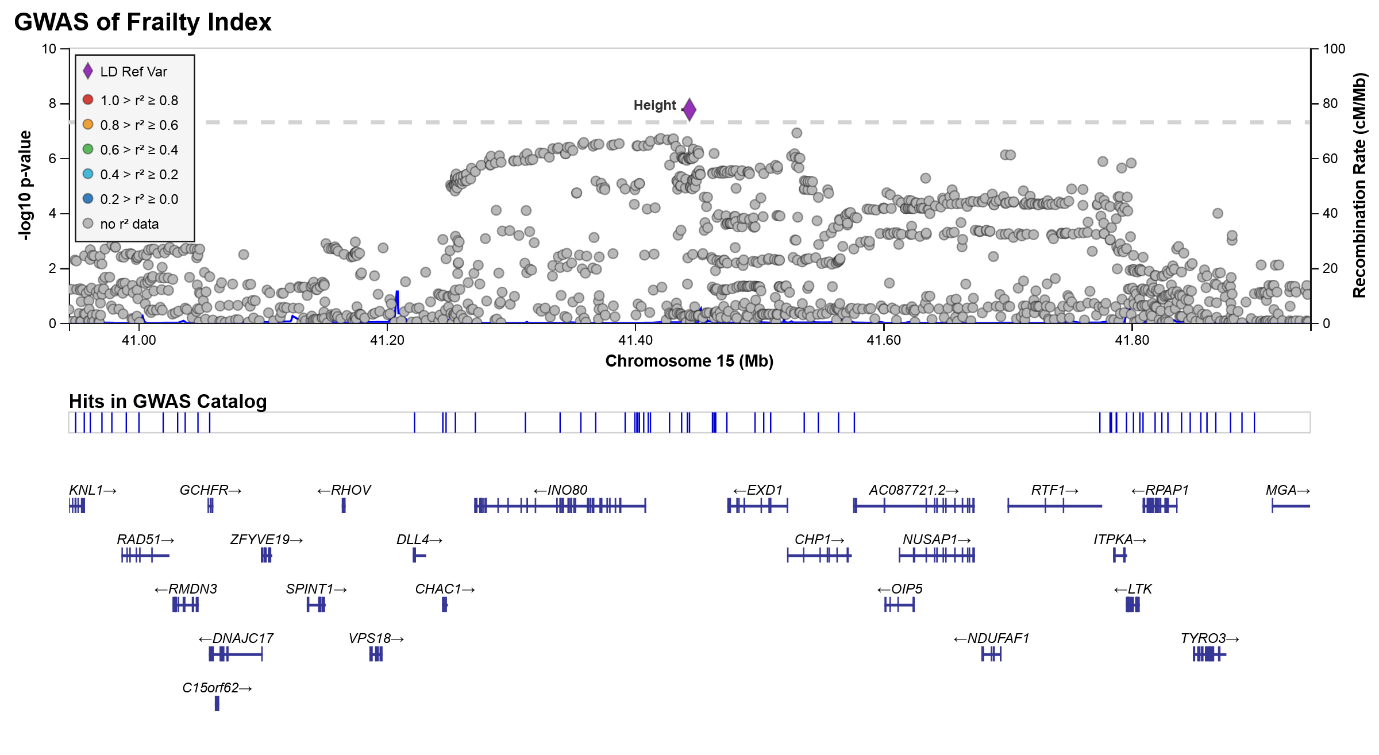


## Supplementary Figure 2M: rs17612102 (15:52264094:T:C)


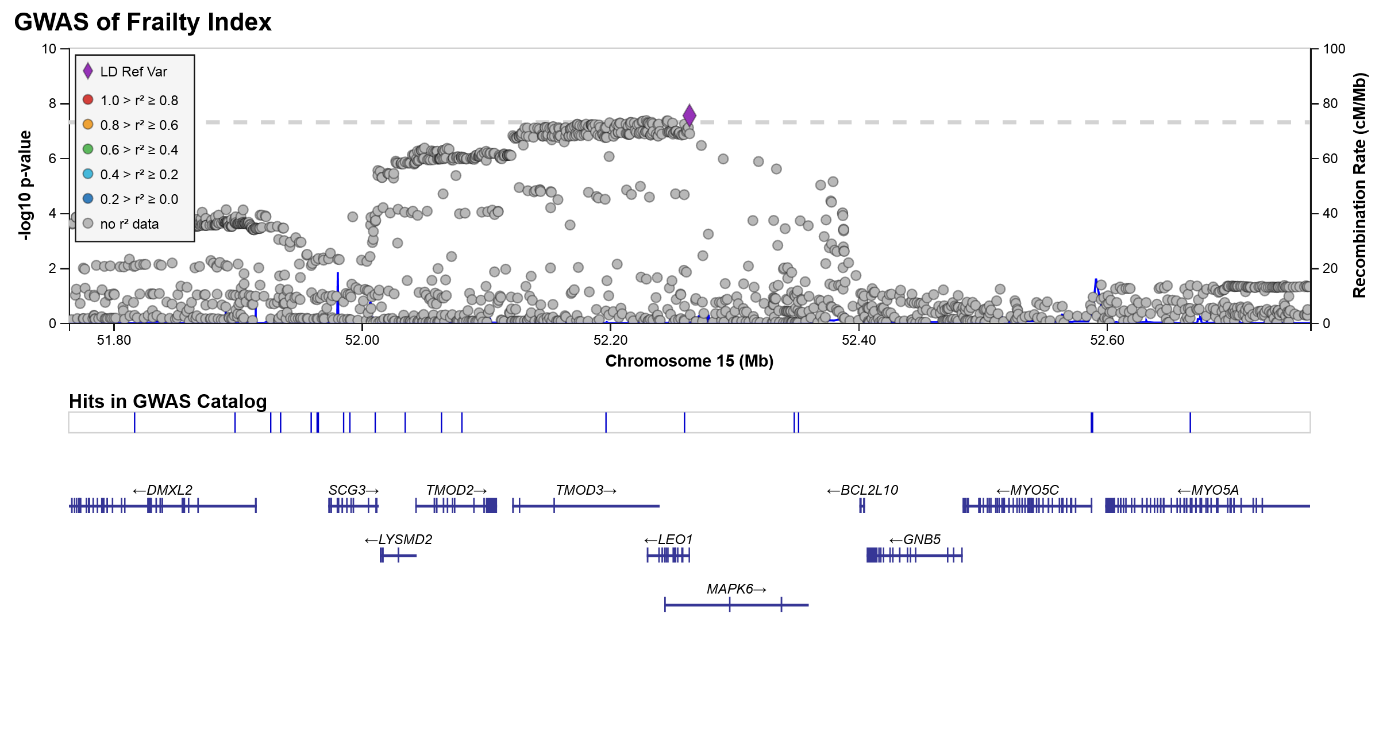


## Supplementary Figure 2N: rs8089807 (18:39322639:T:C)


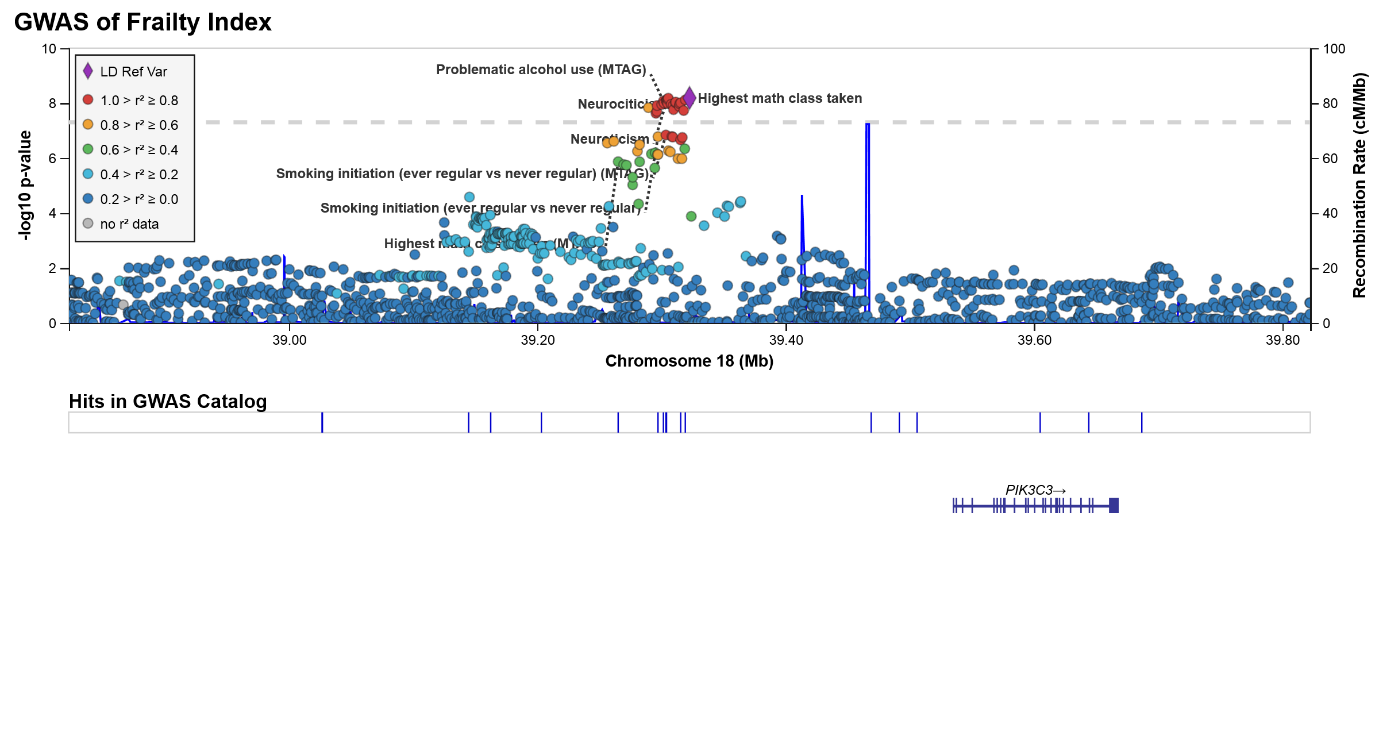


## Supplementary Figure 3: Mendelian randomization: estimated effect of higher BMI on the frailty index in UK Biobank


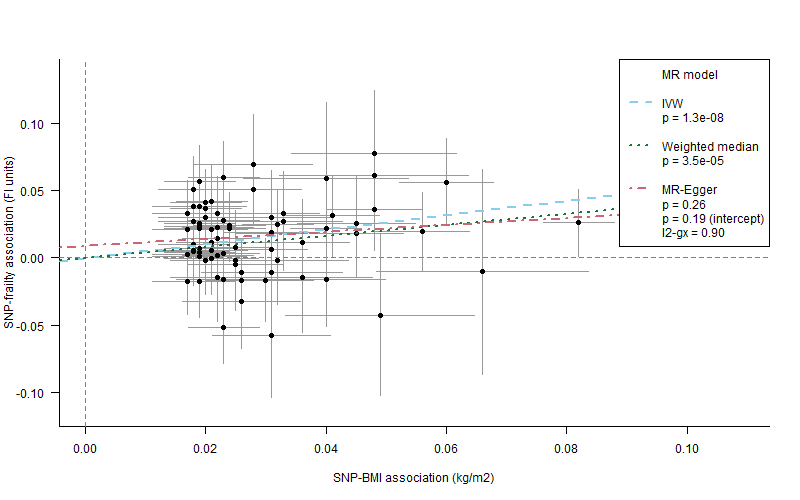


## Supplementary Figure 4: Mendelian randomization: estimated effect of a higher liability to inflammatory bowel disease on the frailty index in UK Biobank


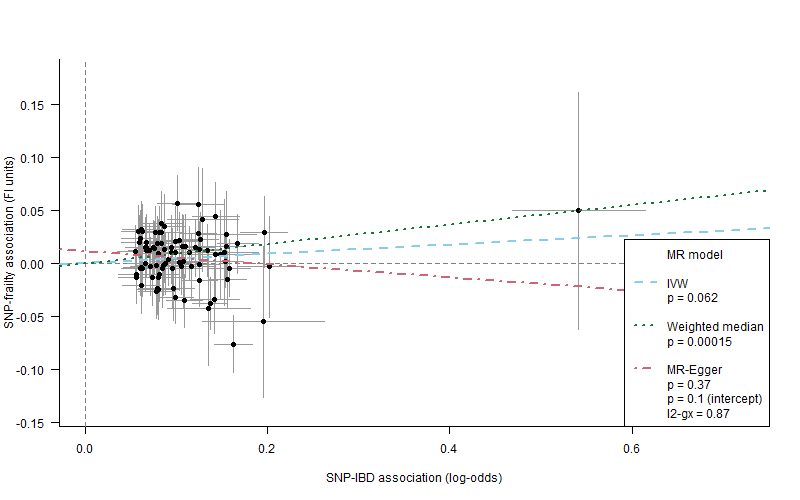


## Supplementary Figure 5: Mendelian randomization: estimated effect of a higher waist-to-hip ratio on the frailty index in UK Biobank


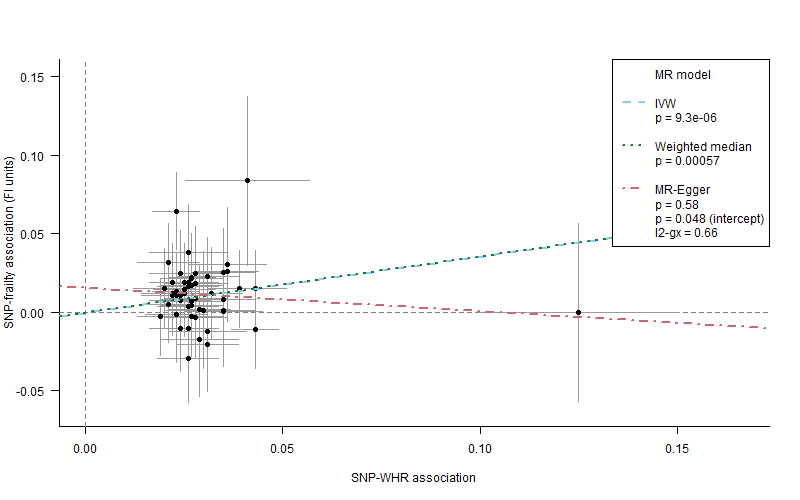


## Supplementary Figure 6: Mendelian randomization: estimated effect of liability for a higher age at menarche on the frailty index in UK Biobank


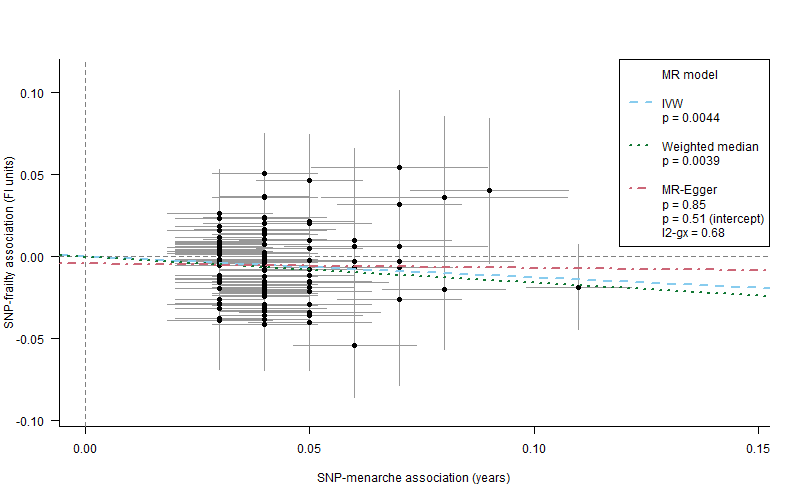


## Supplementary Figure 7: Mendelian randomization: estimated effect of higher grip strength on the frailty index in UK Biobank


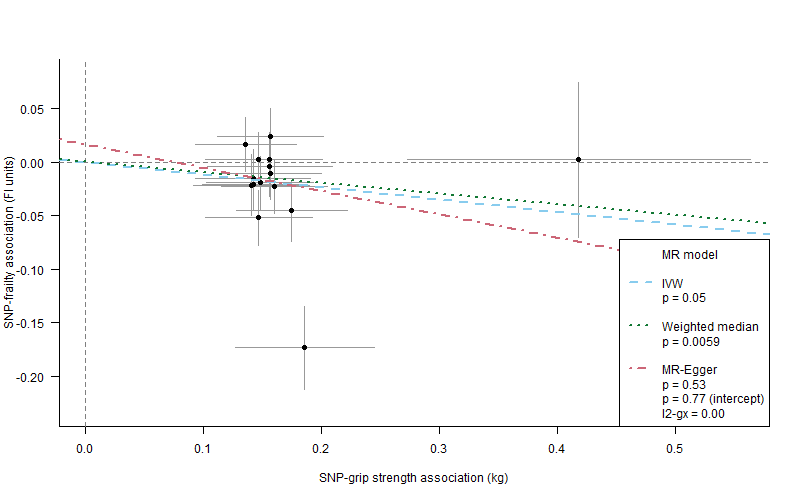


## Supplementary Figure 8: Mendelian randomization: estimated effect of liability for a higher age at first sexual intercourse on the frailty index in UK Biobank


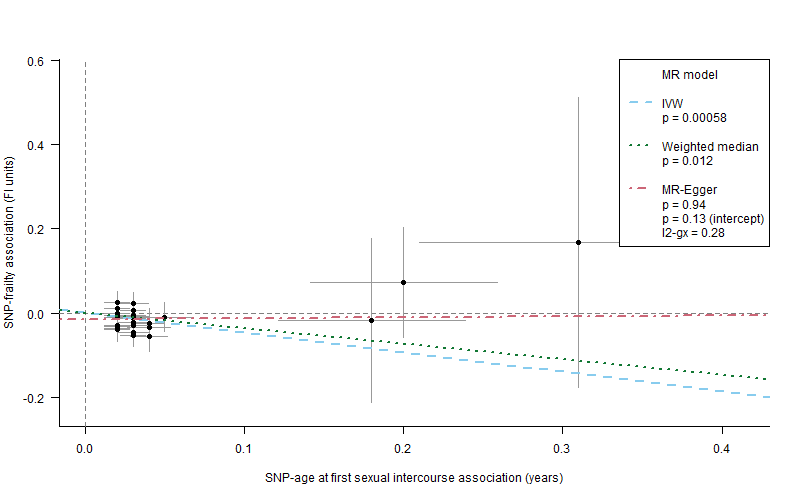


Two-sample Mendelian randomization analysis of age at first sexual intercourse-associated variants on Frailty Index.

## Supplementary Figure 9: Mendelian randomization: estimated effect of higher parental survival on the frailty index in UK Biobank


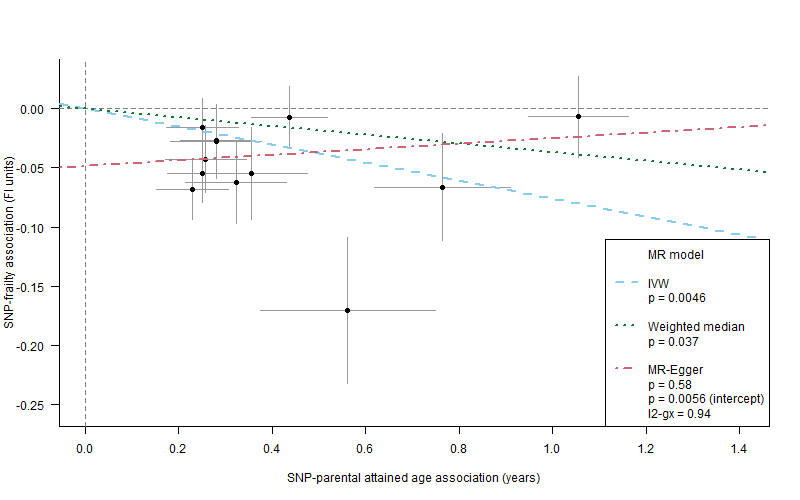


# References

Ardlie KG, DeLuca DS, Segrè A V., Sullivan TJ, Young TR, Gelfand ET, Trowbridge CA, Maller JB, Tukiainen T, Lek M, Ward LD, Kheradpour P, Iriarte B, Meng Y, Palmer CD, Esko T, Winckler W, Hirschhorn JN, Kellis M, MacArthur DG, Getz G, Shabalin AA, Li G, Zhou YH, Nobel AB, Rusyn I, Wright FA, Lappalainen T, Ferreira PG, Ongen H, Rivas MA, Battle A, Mostafavi S, Monlong J, Sammeth M, Melé M, Reverter F, Goldmann JM, Koller D, Guigó R, McCarthy MI, Dermitzakis ET, Gamazon ER, Im HK, Konkashbaev A, Nicolae DL, Cox NJ, Flutre T, Wen X, Stephens M, Pritchard JK, Tu Z, Zhang B, Huang T, Long Q, Lin L, Yang J, Zhu J, Liu J, Brown A, Mestichelli B, Tidwell D, Lo E, Salvatore M, Shad S, Thomas JA, Lonsdale JT, Moser MT, Gillard BM, Karasik E, Ramsey K, Choi C, Foster BA, Syron J, Fleming J, Magazine H, Hasz R, Walters GD, Bridge JP, Miklos M, Sullivan S, Barker LK, Traino HM, Mosavel M, Siminoff LA, Valley DR, Rohrer DC, Jewell SD, Branton PA, Sobin LH, Barcus M, Qi L, McLean J, Hariharan P, Um KS, Wu S, Tabor D, Shive C, Smith AM, Buia SA, Undale AH, Robinson KL, Roche N, Valentino KM, Britton A, Burges R, Bradbury D, Hambright KW, Seleski J, Korzeniewski GE, Erickson K, Marcus Y, Tejada J, Taherian M, Lu C, Basile M, Mash DC, Volpi S, Struewing JP, Temple GF, Boyer J, Colantuoni D, Little R, Koester S, Carithers LJ, Moore HM, Guan P, Compton C, Sawyer SJ, Demchok JP, Vaught JB, Rabiner CA & Lockhart (2015) The Genotype-Tissue Expression (GTEx) pilot analysis: Multitissue gene regulation in humans. *Science (80-. ).* 348, 648–660.

Bonder MJ, Luijk R, Zhernakova D V., Moed M, Deelen P, Vermaat M, Van Iterson M, Van Dijk F, Van Galen M, Bot J, Slieker RC, Jhamai PM, Verbiest M, Suchiman HED, Verkerk M, Van Der Breggen R, Van Rooij J, Lakenberg N, Arindrarto W, Kielbasa SM, Jonkers I, Van’t Hof P, Nooren I, Beekman M, Deelen J, Van Heemst D, Zhernakova A, Tigchelaar EF, Swertz MA, Hofman A, Uitterlinden AG, Pool R, Van Dongen J, Hottenga JJ, Stehouwer CDA, Van Der Kallen CJH, Schalkwijk CG, Van Den Berg LH, Van Zwet EW, Mei H, Li Y, Lemire M, Hudson TJ, Slagboom PE, Wijmenga C, Veldink JH, Van Greevenbroek MMJ, Van Duijn CM, Boomsma DI, Isaacs A, Jansen R, Van Meurs JBJ, Hoen’t PAC, Franke L & Heijmans BT (2017) Disease variants alter transcription factor levels and methylation of their binding sites. *Nat. Genet.* 49, 131–138.

Bowden J, Fabiola Del Greco M, Minelli C, Smith GD, Sheehan NA & Thompson JR (2016) Assessing the suitability of summary data for two-sample mendelian randomization analyses using MR-Egger regression: The role of the I2statistic. *Int. J. Epidemiol.* 45, 1961–1974.

Broad Institute RICOPILI: Rapid Imputation and COmputational PIpeLIne for Genome-Wide Association Studies.

Bulik-Sullivan BK, Loh P-R, Finucane HK, Ripke S, Yang J, Consortium SWG of the PG, Patterson N, Daly MJ, Price AL & Neale BM (2015) LD Score regression distinguishes confounding from polygenicity in genome-wide association studies. *Nat Genet* advance on, 291–295.

Burgess S, Bowden J, Fall T, Ingelsson E & Thompson SG (2017) Sensitivity analyses for robust causal inference from mendelian randomization analyses with multiple genetic variants. *Epidemiology*.

Chen L, Ge B, Casale FP, Vasquez L, Kwan T, Garrido-Martín D, Watt S, Yan Y, Kundu K, Ecker S, Datta A, Richardson D, Burden F, Mead D, Mann AL, Fernandez JM, Rowlston S, Wilder SP, Farrow S, Shao X, Lambourne JJ, Redensek A, Albers CA, Amstislavskiy V, Ashford S, Berentsen K, Bomba L, Bourque G, Bujold D, Busche S, Caron M, Chen SH, Cheung W, Delaneau O, Dermitzakis ET, Elding H, Colgiu I, Bagger FO, Flicek P, Habibi E, Iotchkova V, Janssen-Megens E, Kim B, Lehrach H, Lowy E, Mandoli A, Matarese F, Maurano MT, Morris JA, Pancaldi V, Pourfarzad F, Rehnstrom K, Rendon A, Risch T, Sharifi N, Simon MM, Sultan M, Valencia A, Walter K, Wang SY, Frontini M, Antonarakis SE, Clarke L, Yaspo ML, Beck S, Guigo R, Rico D, Martens JHA, Ouwehand WH, Kuijpers TW, Paul DS, Stunnenberg HG, Stegle O, Downes K, Pastinen T & Soranzo N (2016) Genetic Drivers of Epigenetic and Transcriptional Variation in Human Immune Cells. *Cell* 167, 1398–1414.e24.

Finucane HK, Reshef YA, Anttila V, Slowikowski K, Gusev A, Byrnes A, Gazal S, Loh PR, Lareau C, Shoresh N, Genovese G, Saunders A, Macosko E, Pollack S, Perry JRB, Buenrostro JD, Bernstein BE, Raychaudhuri S, McCarroll S, Neale BM & Price AL (2018) Heritability enrichment of specifically expressed genes identifies disease-relevant tissues and cell types. *Nat. Genet.* 50, 621–629.

Gaunt TR, Shihab HA, Hemani G, Min JL, Woodward G, Lyttleton O, Zheng J, Duggirala A, McArdle WL, Ho K, Ring SM, Evans DM, Davey Smith G & Relton CL (2016) Systematic identification of genetic influences on methylation across the human life course. *Genome Biol.* 17, 61.

Howie BN, Donnelly P & Marchini J (2009) A Flexible and Accurate Genotype Imputation Method for the Next Generation of Genome-Wide Association Studies N. J. Schork, ed. *PLoS Genet.* 5, e1000529.

Kamat MA, Blackshaw JA, Young R, Surendran P, Burgess S, Danesh J, Butterworth AS & Staley JR (2019) PhenoScanner V2: an expanded tool for searching human genotype-phenotype associations. *Bioinformatics*, 1–3.

de Leeuw CA, Mooij JM, Heskes T & Posthuma D (2015) MAGMA: Generalized Gene-Set Analysis of GWAS Data. *PLoS Comput. Biol.* 11.

Loh P-R, Danecek P, Palamara PF, Fuchsberger C, A Reshef Y, K Finucane H, Schoenherr S, Forer L, McCarthy S, Abecasis GR, Durbin R & L Price A (2016) Reference-based phasing using the Haplotype Reference Consortium panel. *Nat. Genet.* 48, 1443–1448.

Loh P-R, Tucker G, Bulik-Sullivan BK, Vilhjálmsson BJ, Finucane HK, Salem RM, Chasman DI, Ridker PM, Neale BM, Berger B, Patterson N & Price AL (2015) Efficient Bayesian mixed-model analysis increases association power in large cohorts. *Nat. Genet.* 47, 284–90.

Magnusson PKE, Almqvist C, Rahman I, Ganna A, Viktorin A, Walum H, Halldner L, Lundström S, Ullén F, Långström N, Larsson H, Nyman A, Gumpert CH, Råstam M, Anckarsäter H, Cnattingius S, Johannesson M, Ingelsson E, Klareskog L, de Faire U, Pedersen NL & Lichtenstein P (2013) The Swedish Twin Registry: establishment of a biobank and other recent developments. *Twin Res. Hum. Genet.* 16, 317–29.

McCarthy S, Das S, Kretzschmar W, Delaneau O, Wood AR, Teumer A, Kang HM, Fuchsberger C, Danecek P, Sharp K, Luo Y, Sidore C, Kwong A, Timpson N, Koskinen S, Vrieze S, Scott LJ, Zhang H, Mahajan A, Veldink J, Peters U, Pato C, van Duijn CM, Gillies CE, Gandin I, Mezzavilla M, Gilly A, Cocca M, Traglia M, Angius A, Barrett JC, Boomsma D, Branham K, Breen G, Brummett CM, Busonero F, Campbell H, Chan A, Chen S, Chew E, Collins FS, Corbin LJ, Smith GD, Dedoussis G, Dorr M, Farmaki A-E, Ferrucci L, Forer L, Fraser RM, Gabriel S, Levy S, Groop L, Harrison T, Hattersley A, Holmen OL, Hveem K, Kretzler M, Lee JC, McGue M, Meitinger T, Melzer D, Min JL, Mohlke KL, Vincent JB, Nauck M, Nickerson D, Palotie A, Pato M, Pirastu N, McInnis M, Richards JB, Sala C, Salomaa V, Schlessinger D, Schoenherr S, Slagboom PE, Small K, Spector T, Stambolian D, Tuke M, Tuomilehto J, Van den Berg LH, Van Rheenen W, Volker U, Wijmenga C, Toniolo D, Zeggini E, Gasparini P, Sampson MG, Wilson JF, Frayling T, de Bakker PIW, Swertz MA, McCarroll S, Kooperberg C, Dekker A, Altshuler D, Willer C, Iacono W, Ripatti S, Soranzo N, Walter K, Swaroop A, Cucca F, Anderson CA, Myers RM, Boehnke M, McCarthy MI, Durbin R & Haplotype Reference Consortium (2016) A reference panel of 64,976 haplotypes for genotype imputation. *Nat. Genet.* 48, 1279–83.

Sun BB, Maranville JC, Peters JE, Stacey D, Staley JR, Blackshaw J, Burgess S, Jiang T, Paige E, Surendran P, Oliver-Williams C, Kamat MA, Prins BP, Wilcox SK, Zimmerman ES, Chi A, Bansal N, Spain SL, Wood AM, Morrell NW, Bradley JR, Janjic N, Roberts DJ, Ouwehand WH, Todd JA, Soranzo N, Suhre K, Paul DS, Fox CS, Plenge RM, Danesh J, Runz H & Butterworth AS (2018) Genomic atlas of the human plasma proteome. *Nature* 558, 73–79.

Võsa U, Claringbould A, Westra H-J, Bonder MJ, Deelen P, Zeng B, Kirsten H, Saha A, Kreuzhuber R, Kasela S, Pervjakova N, Alvaes I, Fave M-J, Agbessi M, Christiansen M, Jansen R, Seppälä I, Tong L, Teumer A, Schramm K, Hemani G, Verlouw J, Yaghootkar H, Sönmez R, Brown A, Kukushkina V, Kalnapenkis A, Rüeger S, Porcu E, Kronberg-Guzman J, Kettunen J, Powell J, Lee B, Zhang F, Arindrarto W, Beutner F, Brugge H, Dmitreva J, Elansary M, Fairfax BP, Georges M, Heijmans BT, Kähönen M, Kim Y, Knight JC, Kovacs P, Krohn K, Li S, Loeffler M, Marigorta UM, Mei H, Momozawa Y, Müller-Nurasyid M, Nauck M, Nivard M, Penninx B, Pritchard J, Raitakari O, Rotzchke O, Slagboom EP, Stehouwer CDA, Stumvoll M, Sullivan P, Hoen PAC ‘t, Thiery J, Tönjes A, van Dongen J, van Iterson M, Veldink J, Völker U, Wijmenga C, Swertz M, Andiappan A, Montgomery GW, Ripatti S, Perola M, Kutalik Z, Dermitzakis E, Bergmann S, Frayling T, van Meurs J, Prokisch H, Ahsan H, Pierce B, Lehtimäki T, Boomsma D, Psaty BM, Gharib SA, Awadalla P, Milani L, Ouwehand W, Downes K, Stegle O, Battle A, Yang J, Visscher PM, Scholz M, Gibson G, Esko T & Franke L (2018) Unraveling the polygenic architecture of complex traits using blood eQTL metaanalysis. *bioRxiv*, 447367. Available at: http://biorxiv.org/content/early/2018/10/19/447367.abstract.

Wang Y, Karlsson R, Lampa E, Zhang Q, Hedman ÅK, Almgren M, Almqvist C, McRae AF, Marioni RE, Ingelsson E, Visscher PM, Deary IJ, Lind L, Morris T, Beck S, Pedersen NL & Hägg S (2018) Epigenetic influences on aging: a longitudinal genome-wide methylation study in old Swedish twins. *Epigenetics* 13, 975–987.

Watanabe K, Taskesen E, Bochoven A van & Posthuma D (2017) Functional mapping and annotation of genetic associations with FUMA. *bioRxiv*, 110023.

Yavorska OO & Burgess S (2017) MendelianRandomization: An R package for performing Mendelian randomization analyses using summarized data. *Int. J. Epidemiol.* 46, 1734–1739.
